# Nascent Glycoproteome Reveals That N-Linked Glycosylation Inhibitor-1 Suppresses Expression of Glycosylated Lysosome-Associated Membrane Protein-2

**DOI:** 10.3389/fmolb.2022.899192

**Published:** 2022-04-27

**Authors:** Xinyi Cao, Peiyi Meng, Yuyin Shao, Guoquan Yan, Jun Yao, Xinwen Zhou, Chao Liu, Lei Zhang, Hong Shu, Haojie Lu

**Affiliations:** ^1^ Institutes of Biomedical Sciences and Shanghai Cancer Center, Fudan University, Shanghai, China; ^2^ Department of Chemistry, Fudan University, Shanghai, China; ^3^ Beijing Advanced Innovation Center for Precision Medicine, Beihang University, Beijing, China; ^4^ Department of Clinical Laboratory, Guangxi Medical University Cancer Hospital, Nanning, China; ^5^ NHC Key Laboratory of Glycoconjugates Research, Fudan University, Shanghai, China

**Keywords:** hepatocellular carcinoma, nascent proteome, glycosylation, glycoproteome, LAMP2

## Abstract

Glycosylation inhibition has great potential in cancer treatment. However, the corresponding cellular response, protein expression and glycosylation changes remain unclear. As a cell-permeable small-molecule inhibitor with reduced cellular toxicity, N-linked glycosylation inhibitor-1 (NGI-1) has become a great approach to regulate glycosylation in mammalian cells. Here for the first time, we applied a nascent proteomic method to investigate the effect of NGI-1 in hepatocellular carcinoma (HCC) cell line. Besides, hydrophilic interaction liquid chromatography (HILIC) was adopted for the enrichment of glycosylated peptides. Glycoproteomic analysis revealed the abundance of glycopeptides from LAMP2, NICA, and CEIP2 was significantly changed during NGI-1 treatment. Moreover, the alterations of LAMP2 site-specific intact N-glycopeptides were comprehensively assessed. NGI-1 treatment also led to the inhibition of Cathepsin D maturation and the induction of autophagy. In summary, we provided evidence that NGI-1 repressed the expression of glycosylated LAMP2 accompanied with the occurrence of lysosomal defects and autophagy.

## Introduction

The role of glycosylation in modulating various aspects of protein biology is a broad research field (Ward et al., 2021; Critcher et al., 2022). O-linked glycosylation and N-linked glycosylation are the two most important forms of protein glycosylation (Sun et al., 2021). Unlike the O-linked glycosylation, N-linked glycosylation is one type of biological processes that proteins undergo during *de novo* synthesis (Xu et al., 2018; Karki et al., 2021; Tian et al., 2021). N-linked protein glycosylation mainly begins from endoplasmic reticulum (ER). Next, a range of complex enzymatic reactions were carried out with the help of numerous glycosidases, glycosyltransferases, transporters and regulatory proteins located in ER or Golgi apparatus (Groux-Degroote et al., 2021). Well-controlled glycosylation is very important for the maturation and transport of glycoproteins. Once the N-linked glycosylation pathway is interrupted, the unfolded and misfolded glycoproteins are easy to accumulate in the ER, resulting in ER stress (Bull and Thiede, 2012; Di Patria et al., 2022; Wang et al., 2022). Under the condition of continuous stress, the excessive accumulation of abnormally folded proteins in the ER lumen is fatal to cells, which can cause unfolded protein response (UPR) and apoptosis (Sovolyova et al., 2014; Read and Schroder, 2021). However, the detailed response events and underlying molecular mechanisms in N-glycosylation inhibition remain to be elucidated, especially in the fine process of protein *de novo* synthesis.

NGI-1 is developed as a novel inhibitor of N-linked glycosylation with cellular permeability, which can directly target and block the function of the oligosaccharyltransferase (OST) catalytic subunits STT3A and STT3B in the ER (Rinis et al., 2018; Lu et al., 2019; Zhu et al., 2019). Compared with traditional glycosylation inhibitor tunicamycin with obvious toxicity, NGI-1 does not cause complete inhibition of glycosylation and has low cytotoxicity (Dawson et al., 2007; Contessa et al., 2010; Alymova et al., 2022). Notably, the development of NGI-1 provides a pharmacological method to mildly regulate N-linked glycosylation in mammalian cells (Baro et al., 2019; Shu et al., 2019). The clinical value of NGI-1 in the application of oncologic therapies has recently attracted people’s attention. In lung cancer cells, NGI-1 selectively inhibits the proliferation of epidermal growth factor receptor (EGFR)-dependent cell lines by destroying the glycosylation and cell surface localization of EGFR. Many results confirmed that inhibition of glycosylation could be a promising method for the treatment of receptor tyrosine kinase-dependent cancers (Lopez-Sambrooks et al., 2016). Also, the addition of NGI-1 to tyrosine kinase inhibitor therapy is fatal to drug-resistant cell lines (Lopez Sambrooks et al., 2016). Besides, NGI-1 can sensitize multiple models of neoplasms to chemotherapy or radiation, and may overcome the limited success of conventional strategies (Wahl and Lawrence, 2019; Phillips et al., 2022). The mechanisms involved need to be further investigated to promote the clinical application of NGI-1 in tumor therapy.

Although some studies have analyzed the dynamic protein response under traditional glycosylation inhibitor treatment (Xiao et al., 2016; Shu et al., 2019), our study focused on qualitative and quantitative analyses of newly synthesized proteins. Because the nascent sub-proteome is the first to respond to perturbations in theory. Through the bioorthogonal-chemistry-based enrichment strategy, the non nascent proteome is removed, and the remaining newly synthesized proteins can be identified and quantified more easily in mass spectrometry due to the eliminated interference of non nascent proteome. Moreover, to quantify the changes of newly synthesized proteins is more reasonable to attribute the changed results to the changed condition (McClatchy et al., 2018; van Bergen et al., 2022).

Here, we performed a bioorthogonal non-canonical amino-acid tagging (BONCAT)-based quantitative proteomics method for identification and quantitation of newly synthesized proteins under the treatment of NGI-1 to enhance our mechanistic understanding of N-linked glycosylation inhibition process (Dieterich et al., 2007; Shah et al., 2022). According to the following bioinformatic analyses, the functions of the differentially expressed proteins were annotated. Then, the glycoproteome analysis was applied to identify the *de novo* synthesized glycoproteins in response to N-glycosylation inhibition. Functional experiments were performed to confirm the alteration of lysosome-associated membrane protein-2 (LAMP2) under the condition of glycosylation inhibition. Our study demonstrated that NGI-1 had an impact on a wide range of biological processes by regulating protein glycosylation, providing novel insights in explaining its antitumor mechanism.

## Materials and Methods

### Cell Culture and NGI-1 Treatment

The Huh7 and HCCLM3 cells were obtained from the Chinese Academy of Sciences. Cell culture was performed using Dulbecco’s modified Eagle’s medium (DMEM, Gibco) supplemented with 10% fetal bovine serum (FBS, Biological Industries). Briefly, cells were plated for 48 h prior to incubation with various doses or temporal gradients of NGI-1 (MCE). Briefly, 10 mM NGI-1 was dissolved in 1 ml DMSO to create a storage solution, and further diluted to 5–50 μM with culture medium. The same dosage of DMSO was used as a control. Following NGI-1 treatment, the cells were washed with phosphate buffered saline (PBS, Hyclone) and lysed on ice with RIPA buffer (Beyotime) containing Roche protease inhibitor cocktail. Then, the homogenate was sonicated for 30s and centrifuged at 4°C for 10 min at 18,000×g.

### Chemical Metabolic Labeling

Cells were cultured to about 70–80% confluence and treated with l-methionine-free DMEM (Gibco, 21013024) and dialyzed FBS (Gibco) for half an hour. AHA (Cambridge Isotope Laboratories) at 500 μM was added in the absence or presence of 10 μM NGI-1 for 24 h. At the end of incubation, cells were collected and lysed with 0.5% Sodium dodecyl sulfate (SDS, Sigma-Aldrich) containing Roche protease inhibitor cocktail. The concentration of extracted protein was measured by BCA assay (Beyotime).

### Click Reaction

After metabolic labeling by AHA, a click reaction was performed as previously published (Dieterich et al., 2007). Briefly, a cocktail of 500 μM alkyne-biotin reagent (Sigma-Aldrich), 1 mM Copper Sulfate (CuSO_4,_ Sigma-Aldrich), 6 mM 3-(4-((bis((1-tert-butyl-1H-1,2,3-triazol-4-yl)methyl)amino)methyl)-1H-1,2,3-triazol-1-yl)propanol (BTTP, Click Chemistry Tools) and 6 mM Ascorbate (Sigma-Aldrich) was added to 500 μg cell lysate. The samples were incubated for 3 hours at room temperature.

### Enrichment of Newly Synthesized Proteins

Newly synthesized proteins were enriched as previously described (Ma et al., 2017; Ma et al., 2018) with minor modifications. The enrichment was conducted with NeutrAvidin Agarose Resins (GenScript). The beads were washed by 6 M urea (Sigma), 20% ACN and PBS respectively. After reconstitution, the beads were reduced with 10 mM dithiothreitol (DTT) and alkylated with 25 mM iodoacetamide (IAA). Sequencing-grade modified trypsin (Promega) was utilized for on-beads digestion. The obtained peptides were desalted by Zip-Tip (Millipore) and stored at -20°C until use. For glycopeptide enrichment, the Glycopeptide Enrichment Kit (Novagen, Darmstadt, Germany) was used following the instructions.

### LC-MS/MS

The desalted peptides were analyzed on Orbitrap Exploris 480 MS. Peptides were separated by a C18 column (75 μm × 500 mm column, ThermoFisher) on an Easy nLC 1,200 high-pressure liquid chromatography (HPLC) system (Thermo Fisher Scientific) operating at 300 nL/min. Buffer A (0.1% formic acid) and buffer B (0.1% formic acid in 80% ACN) were used. Peptides were separated by a linear gradient from 8% B to 23% B in 90 min followed by a linear increase to 50% B in 27 min. For data-dependent acquisition experiments, the full MS resolutions were set to 120,000 and full MS AGC target was 300%. Mass range was set to 350–1,200 Da. AGC target value for fragment spectra was set at 75% with a resolution of 15,000. Isolation width was set at 1.6 m/z. Precursor ions were fragmented by higher energy collisional dissociation with a normalized collision energy of 29%. All data were acquired in positive polarity profile mode. For intact glycopeptide analysis, the experimental instrument and conditions were described previously with minor modification (Zhang et al., 2019; Cao et al., 2021). To ensure equal amount of analyte loading, the same volume (4 μl) was loaded onto the trap column (PepMap C18, 100 μm × 2 cm) and the analytical column (PepMap C18, 75 μm × 25 cm) by using a gradient of 1–25% solvent B (solvent A: water with 0.1% FA; solvent B: ACN with 0.1% FA) in 60 min, followed by an increase to 45% B in 20 min.

### Data Analysis

Peptide identification and label-free relative quantification analysis was performed in PEAKS Studio software (Bioinformatics Solutions Inc., Waterloo, Canada). PEAKS provided an unbiased quantitative workflow based on the MS1 peak area without internal standard. The protein database was from Swiss-Prot reviewed, date July 2020, with species of Homo sapiens (20,380 entries). The input parameters were: 10 ppm precursor mass tolerance, 0.02 Da fragment mass tolerance. The maximum false discovery rates for protein and peptides were set at 1%. Gene ontology (GO) analysis, including cellular component (CC), molecular function (MF), and biological process (BP), was performed through the David Functional Annotation Tool (Huang da et al., 2009). The intact glycopeptides were identified by pGlyco 3.0 (Version 2020.12.08) and quantified by pQuant (Liu et al., 2014; Zeng et al., 2021). Parameters for database search could be found in our previous study (Zhang et al., 2019; Cao et al., 2021). The default setting of 1% FDR at the glycopeptide level was used.

### Western Blotting

The obtained cell proteins were resolved by SDS-PAGE. After transferring, the membranes were blocked in 5% defatted milk (Bio-Rad) and then incubated with the primary antibodies: anti-LAMP2 (1:500 dilution, Santa Cruz Biotechnology, United States), Cathepsin D (1:1,000 dilution, Cell Signaling Technology, United States), LC3B (1:1,000 dilution, Cell Signaling Technology, United States), ATG5 (1:1,000 dilution, Proteintech, United States) and β-actin (1:10,000 dilution, Multisciences, China) overnight at 4°C, followed by HRP-conjugated secondary antibodies (Multisciences, China). Immunoblots were visualized by ECL substrate (Biosharp) and Bio-Rad Image Lab software.

### Lysosome Isolation and Visualization

Lysosomes were isolated by using Minute™ Lysosome Isolation Kit (Invent Biotechnologies, United States) according to instructions. For visualization of lysosomes, 50 nM LysoTracker Red (Beyotime) was added to culture medium for 1 hour incubation. After fixation with 4% paraformaldehyde, the cells were stained with DAPI (Keygen Biotech). Olympus fluorescence microscopy was used for image acquisition.

### Transient Transfection and Cell Viability Assay

The small interfering RNA (siRNA) targeting human ATG5 was commercially synthesized (Sangon Biotech) as described (Shi et al., 2021). Along with scrambled RNAi oligonucleotides, the siRNA targeting AGT5 was transfected into cells via transfection reagent (BBI). Cell Counting Kit-8 (CCK8, Dojindo) was performed to confirm the influence of NGI-1 on cell viability and was repeated at least three times.

### Statistical Analysis

Figures were plotted with GraphPad Prism (GraphPad Software Inc.) and Hiplot (https://hiplot.com.cn). The numeric data were showed as means ± standard deviation. Two tailed Student t-test was used for statistical comparison. *p* < 0.05 was considered statistically significant. N-glycan structures were drawn with GlycoWorkbench.

## Results

### Analysis of *de novo* Synthesized Proteins Under Glycosylation Inhibition

**FIGURE 1:**
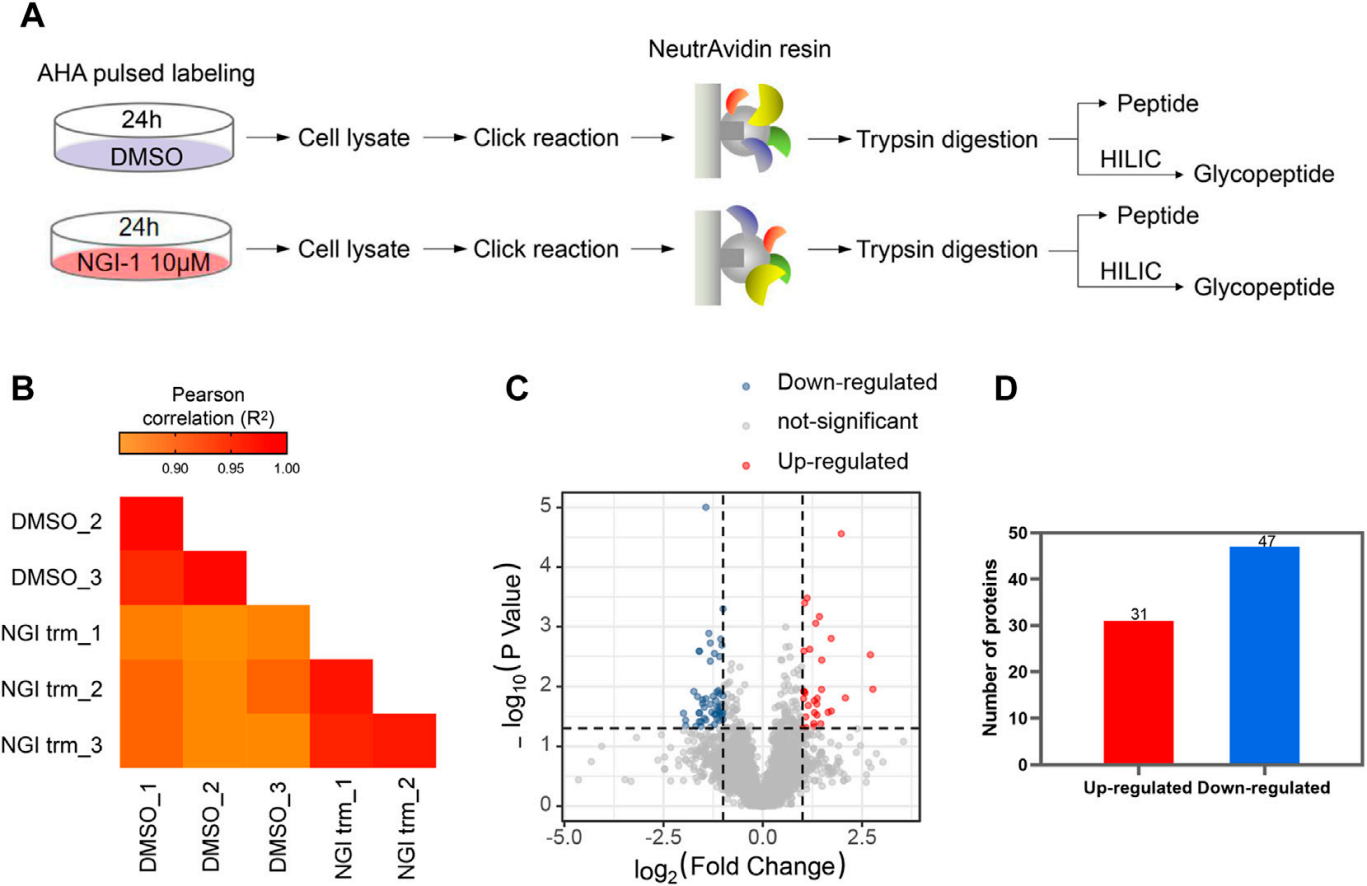
Analysis of nascent proteome under glycosylation inhibition **(A)** Workflow for metabolic labeling of *de novo* protein synthesis in NGI-1 treatment **(B)** The Pearson Correlation Coefficient *R* ^2^ values in technical repetition **(C)** Proteins with statistical significance after a *t*-test were shown in red (up-regulated) or blue (down-regulated) on a volcano plot. The vertical lines correspond to 2.0-fold up and down (ratio between NGI treatment and DMSO), and the horizontal line represents a *p*-value of 0.05. Each point in the plot represents a different protein **(D)** A total of 31 up-regulated and 47 down-regulated *de novo* synthesized proteins were identified.

**FIGURE 2:**
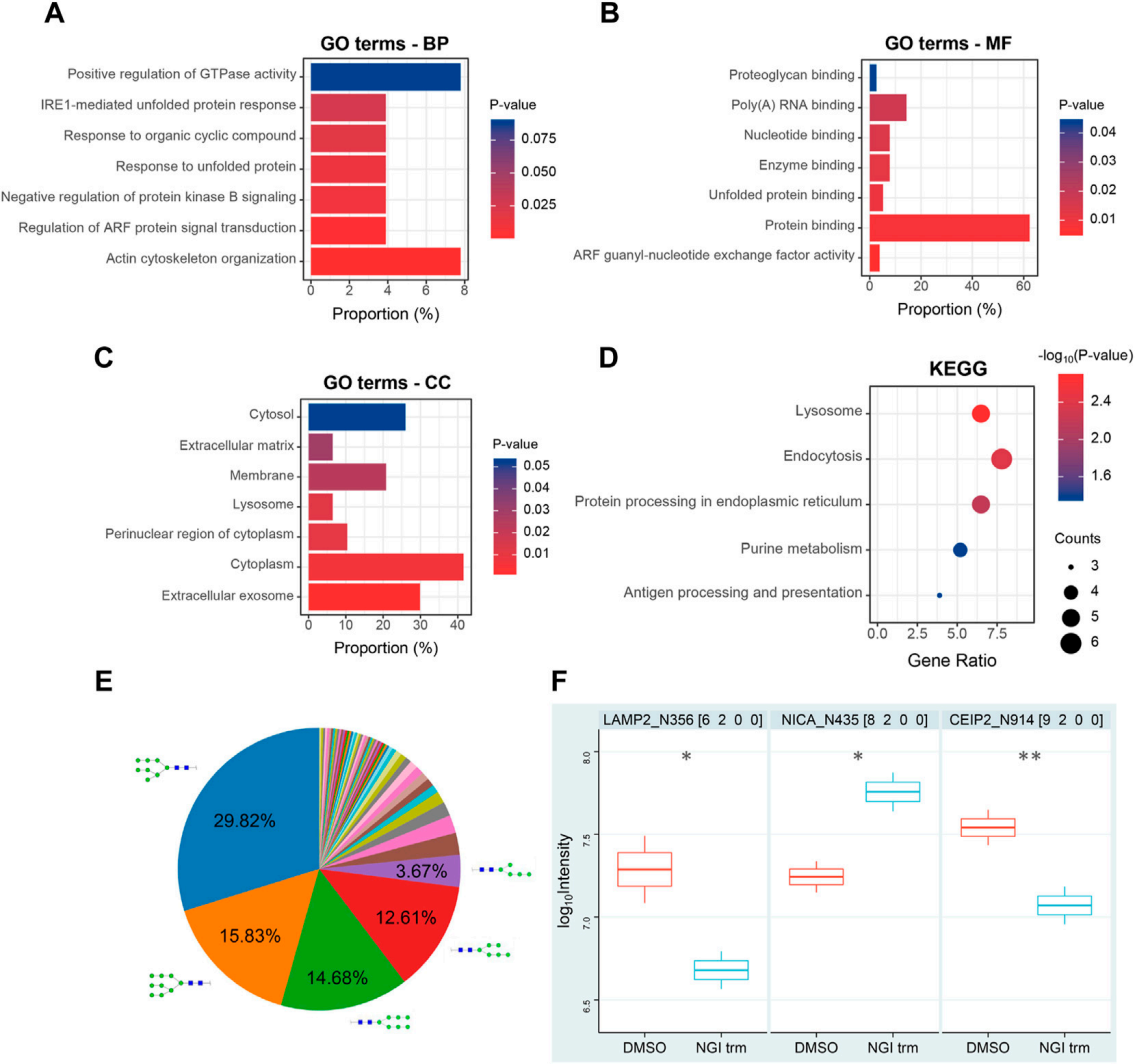
Bioinformatic analysis of nascent proteome **(A–C)** GO biological process (BP), molecular function (MF), and cellular component (CC) categories enriched during N-glycosylation inhibition **(D)** KEGG pathway analysis of differentially expressed *de novo* synthesized proteins **(E)** Distribution of the identified N-glycan compositions **(F)** Differentially expressed *de novo* synthesized intact glycopeptides in Huh7 cells upon NGI-1 treatment. *p* values were determined using two-tailed t-tests. *p < 0.05, **p < 0.01. The number after the protein name represents N-glycosylation site.

For comprehensive characterization of the protein alterations under glycosylation inhibition, we applied the small-molecule OST inhibitor NGI-1 to the *de novo* protein synthesis process (Figure 1A). After starvation, the cells were treated with AHA to specifically label *de novo* synthesized proteins for 24 h under 10 μM NGI-1 treatment or DMSO. Then the cells were harvested, lysed and reacted with alkyne-biotin, followed by enrichment via biotin-avidin affinity interaction in parallel. After incubation and thorough washing, the bound proteins were digested on beads. Ten-percent enzymolysis products were utilized for desalination, and the rest were used for glycopeptide enrichment (Figure 1A). The obtained peptides and glycopeptides were sent to LC-MS/MS for further identification and quantification. In the proteome, 5,508 newly synthesized proteins were identified, of which 4,993 proteins obtained quantitative information. The technical replicates showed good correlation (Figure 1B). As the cutoff criterion was set to fold change (FC, NGI treatment/DMSO) > 2 or <0.5, a total of 78 newly synthesized proteins (31 up- and 47 down-regulated) were differentially expressed (Figures 1C,D; Supplementary Table S1). Actin cytoskeleton organization and protein binding process were most enriched by GO-BP and GO-MF analysis, and the cytoplasm was enriched according to GO-CC analysis (Figures 2A–C). The Kyoto Encyclopedia of Genes and Genomes (KEGG) analysis of the differentially expressed proteins indicated significant enrichment in lysosome-related pathways (Figure 2D).

### Analysis of Newly Synthesized Glycoproteins During NGI-1 Treatment

For convenience, a four-digit nomenclature in HNSF order was used for glycan annotation (H, Hexose; N, N-acetylhexosamine; S, Sialic acid; F, Fucose). In the glycoproteome, 436 newly synthesized N-glycopeptides were identified under the condition of NGI-1 treatment (Supplementary Table S2). Comparing the distribution of N-glycan compositions, H8N2 and H9N2 represented the most common compositions in the identification (Figure 2E). With the help of a quantitation software tool termed pQuant (Liu et al., 2014), the peak area was determined for each glycopeptide. At last 47 N-glycopeptides passed the strict filtering criteria (Zhang et al., 2019; Cao et al., 2021) and obtained quantitative information (Supplementary Table S3). Compared with the control group, LAMP2_N356 (H6N2) and CEIP2_N914 (H9N2) were significantly decreased under NGI-1 treatment (*p* = 0.0434 and *p* = 0.0049, respectively), while NICA_N435 (H8N2) was significantly increased (*p* = 0.0465) (Figure 2F).

### NGI-1 Treatment Inhibited the Expression of Glycosylated LAMP2 and Caused Lysosomal Defects

**FIGURE 3:**
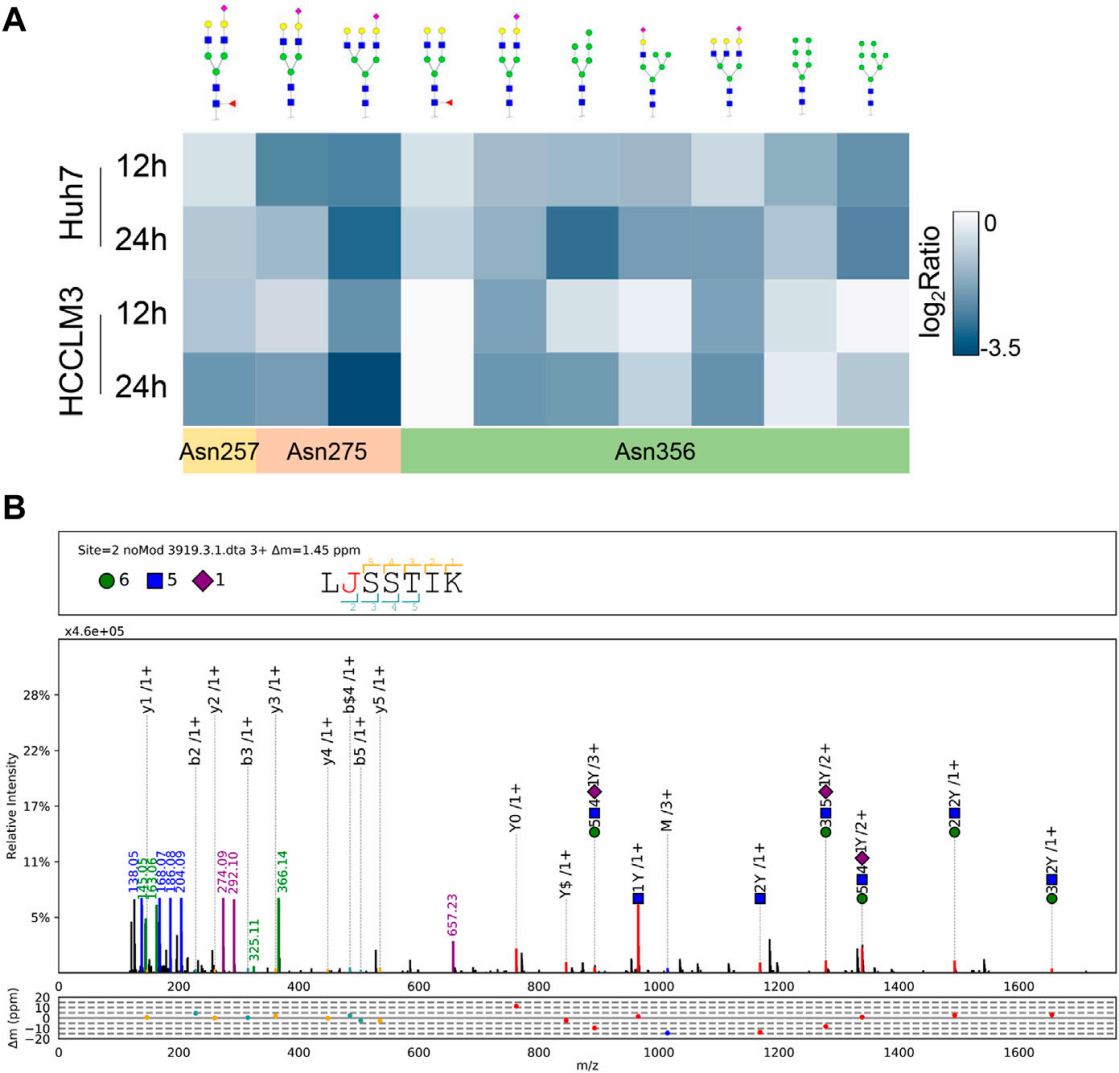
Site-specific glycan profiling of LAMP2 **(A)** Heat map showing site-specific intact glycopeptides from LAMP2 in Huh7 and HCCLM3 cells with NGI-1 treatment. The positions of identified N-glycosylation sites were annotated **(B)** pGlyco annotation of LAMP2_N275 (H6N5S1). “J” represents the glycosylation site “N”; green circle: hexose (H); blue square: N-acetylglucosamine (N); purple rhombus: sialic acid (S). The upper frame of spectrum is designed to annotate peptide sequence and glycan composition. The mass deviations of the annotated peaks are shown in the box below.

**FIGURE 4:**
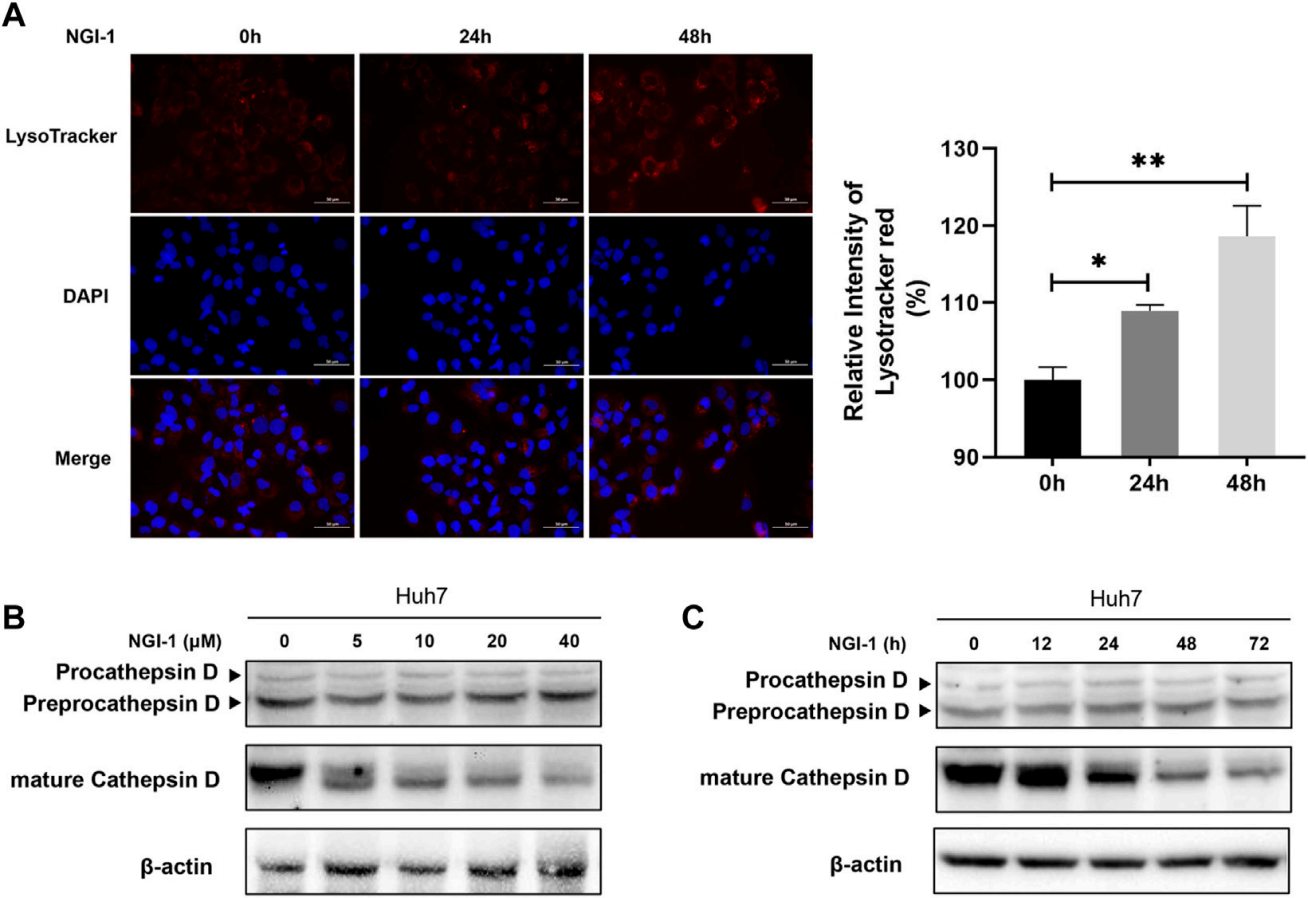
NGI-1 treatment causes lysosomal defects in Huh7 cells. **(A)** Huh7 cells were treated with 10 μM NGI-1 for different time (0, 24, 48 h), followed by labeling with LysoTracker Red and stained with DAPI. Relative intensity of LysoTracker Red was quantified from images (Student t-test; *p < 0.05, **p < 0.01) **(B,C)** Immunoblot analysis of Cathepsin D in Huh7 cells with NGI-1 treatment. Cells were treated either with different concentrations for 24 h **(B)** or for different durations at a concentration of 10 μM **(C)**.

LAMP2 is one of the major proteins on the surface of lysosomes, which is crucial for lysosomal function (Li and Pfeffer, 2016; Liu et al., 2021). As LAMP2 was significantly suppressed in proteome-wide experiment, we first investigated the potential effect of NGI-1 treatment on LAMP2. Western blotting showed that the expression of glycosylated LAMP2 in Huh7 cells was considerably reduced under NGI-1 treatment in a concentration-dependent manner (Supplementary Figure S1). To further confirm the suppression extent of LAMP2 by NGI-1, fine mapping of the N-glycosylation profile of LAMP2 was achieved based on lysosome pre-separation. Moreover, we observed that the top 10 intact glycopeptides of LAMP2 were generally decreased in varying degrees (Figure 3A and Supplementary Table S4). Among them, LAMP2_N275 (H6N5S1) exhibited the most obvious down-regulation (Figure 3B). As illustrated in Figure 4A, prolonged NGI-1 incubation led to the accumulation of lysosomal fluorescence signals. Thus, we hypothesized that glycosylation inhibition caused lysosomal dysfunction. We investigated the maturation of lysosomal protease Cathepsin D, which plays an important role in lysosomal protein degradation (Shi et al., 2021). Western blotting demonstrated that NGI-1 treatment strongly inhibited the cleavage of preprocathepsin D, thereby reducing the production of mature Cathepsin D forms at different concentrations (Figure 4B) and different durations (10 μM) (Figure 4C).

### Suppression of Autophagy Occurrence Promoted NGI-1-Mediated Cytotoxicity

**FIGURE 5:**
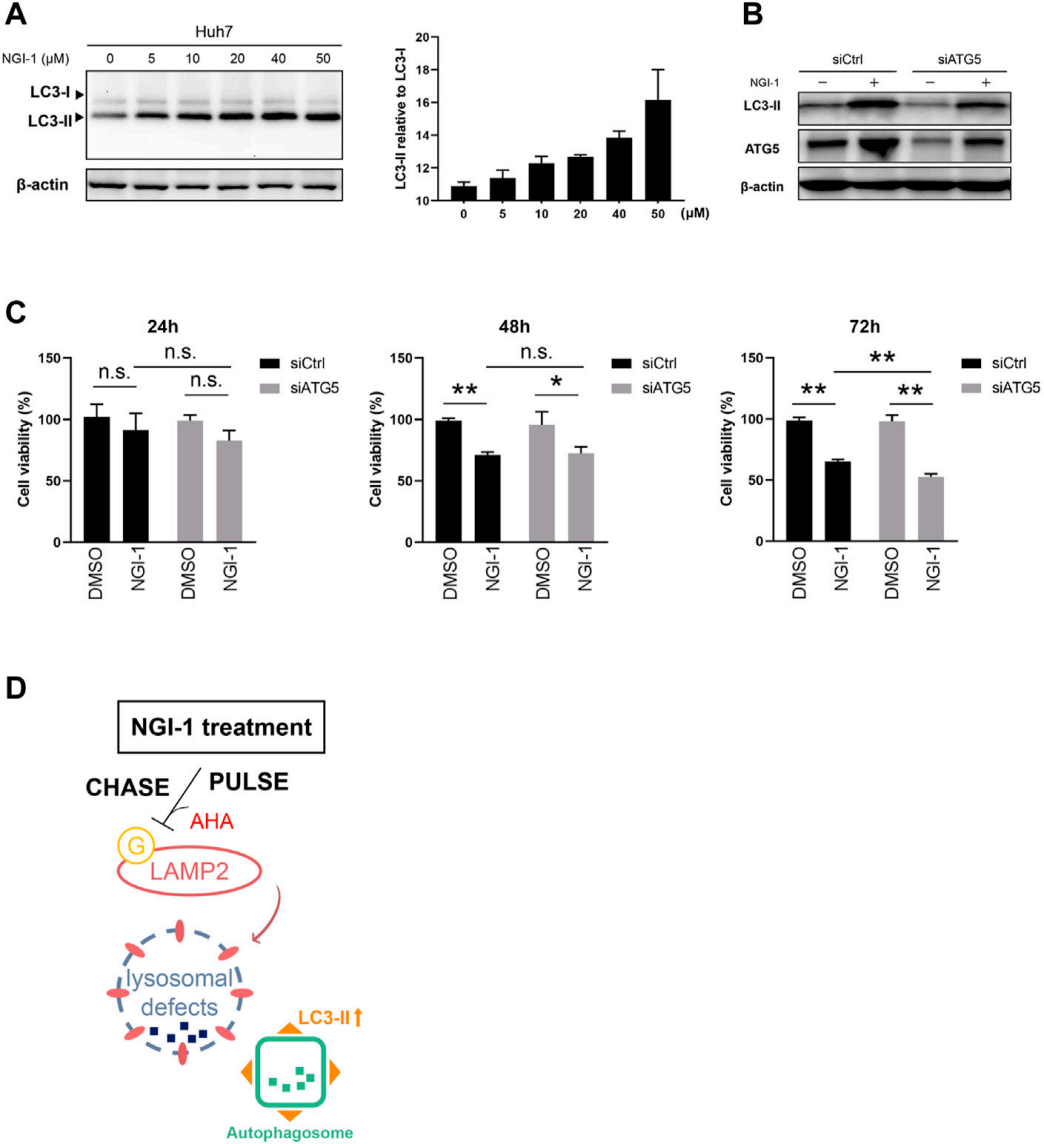
NGI-1 treatment induces autophagy occurrence in Huh7 cells. **(A)** Huh7 cells were treated with NGI-1 at different concentrations and LC3 expression pattern was examined by immunoblot. Endogenous LC3-II/LC3-I ratio was quantified **(B)** Western blotting analysis of LC3 and ATG5 expression in scrambled siRNA- or ATG5 siRNA-treated Huh7 cells incubated with DMSO or NGI-1 (10 μM) for 24 h. Actin was used as the internal control **(C)** Ctrl and ATG5 knockdown Huh7 cells were treated with 10 μM NGI-1 for indicated times and cell viability was determined by Cell Counting Kit-8. Data were presented as Mean ± SD from three independent experiments. *p < 0.05, **p < 0.01, n. s = not significant **(D)** The responses of Huh7 cells toward NGI-1 treatment.

Have shown that NGI-1 treatment disabled LAMP2 by glycosylation inhibition, we therefore hypothesized that NGI-1 was related to chaperone-mediated autophagy which is mediated by LAMP proteins (Hubert et al., 2016; Shi et al., 2021). To solve the problem, we detected the level of autophagy along with NGI-1 treatment. The preliminary results showed that NGI-1 treatment induced a large amount of LC3-II accumulation (Figure 5A). Autophagy is an evolutionarily conserved intracellular process, which maintains homeostasis by degrading and cycling intracellular components in response to various environmental stresses (Cao et al., 2020). Thus, we speculated that the occurrence of autophagy might affect cell viability. To test the hypothesis, the autophagy essential component ATG5 was knocked down to examine the influence of NGI-1-mediated cytotoxicity. Upon NGI-1 treatment, due to the ATG5 knockdown, LC3-II accumulation was considerably decreased (Figure 5B). Under 24 or 48 h treatment, ATG5 knockdown had no effect on NGI-1-mediated decline in cell viability. With the increase of treatment time to 72 h, knocking down of ATG5 exhibited more obvious suppression on cell viability (Figure 5C). The findings suggested that inhibition of autophagy occurrence partially promoted NGI-1-mediated cytotoxicity.

## Discussion

It is widely known that N-linked protein glycosylation is an important cotranslational and post-translational modification in biology, playing an essential part in development, organ specific function and disease (Zielinska et al., 2010; Esmail and Manolson, 2021). Tumor-related glycosylation alterations are connected with tumor progression, malignant transformation and immune evasion (Rodrigues et al., 2018; Bindeman and Fingleton, 2022). In view of the important role of glycosylation in tumor development, glycosylation inhibition has exhibited huge potential as an auxiliary means of antitumor therapies (Song et al., 2020).

Recently, a novel small-molecule OST inhibitor (NGI-1) has provided a pharmacological approach to partially disrupt N-linked glycosylation (Lopez-Sambrooks et al., 2016; Lu et al., 2019; Phillips et al., 2022). In the process of protein synthesis, a unique carbohydrate structure is transferred from a lipid carrier to the -Asn-X-Ser/Thr- (X≠Pro) sequence of the nascent peptides via the OST complex at ER membrane (Aebi, 2013). Therefore, OST represents a critical node for N-glycosylation regulation. Based on this view, NGI-1 was designed to block the function of OST catalytic subunit and induce a selective loss of cell viability (Lopez Sambrooks et al., 2018). However, little is known about *de novo* protein synthesis during the inhibition of glycosylation induced by NGI-1.

Combined with metabolic labeling technology and label-free quantitative proteomics, a workflow was built to characterize the *de novo* protein synthesis in the process of NGI-1-induced glycosylation inhibition. The structure of AHA contains methionine analogues, which enables it to be assembled into *de novo* synthesized proteins. Besides, the azide group of AHA could be used for the enrichment by click reaction (Dieterich et al., 2007; Wang et al., 2016; Vargas-Diaz and Altelaar, 2022). The protein alterations of nascent proteome in NGI-1-treated cells probably reflected specific stress response under N-linked glycosylation inhibition. Our data showed that, the lysosome, endocytosis and ER protein processing related pathways were disturbed as the distinct response to NGI-1 treatment. The discovery of nascent proteome provided a new perspective for comprehending the importance of glycosylation in the growth process of cell. In addition, the advantage of newly synthesized protein enrichment enabled it to be applied in characterizing the glycosylation changes that occurred during protein synthesis, which were usually not detectable. Our glycoproteomic results showed that the alteration of the oligo-mannose glycans was apparently enriched in cancer cells, which was similar to a previous study (Sun et al., 2016). Considering that the increase of high-mannose glycans is a characteristic feature of malignant transformation, this observation indicated that some caution should be used when popularizing the effects of processing inhibitors on a complex process (Li et al., 2015; Boyaval et al., 2022).

It was speculated that when NGI-1 treatment blocks the N-glycosylation synthesis pathway, the changes of related functional glycoproteins may reflect the status of ongoing biological processes. We then focused on a highly glycosylated lysosome membrane protein LAMP2, whose expression of newly synthesized protein and intact glycopeptide were both dramatically down-regulated under NGI-1 treatment. As expected, the overall expression of glycosylated LAMP2 exhibited a similar alteration trend as observed in nascent glycoproteome. LAMP2 is known as a key protein involved in the control of membrane fusion between autophagosome and lysosome. Lysosomal defects usually abolish the fusion of autophagosomes and lysosomes, resulting in excessive accumulation of autophagosomes and occurrence of human diseases (Ikeda et al., 2021; Jiang and Mizushima, 2014). A novel finding in this study was that lysosomal defects occurred along with autophagy accumulation in the NGI-1-exposed Huh7 cells (Figure 5D), suggesting that the fusion of autophagosome-lysosome was blocked up (Liu et al., 2021).

This work highlights the importance of using nascent glycoproteome to comprehensively characterize the targets of glycosylation inhibitors, so that such inhibitors can be better developed and used for basic research or clinical practice. However, due to the complexity of glycoprotein biosynthesis, many studies should be further expanded. For example, more experiments are needed to explore the pathways related to glycosylation inhibition, including glycan biosynthesis. Besides, LAMP2 has different degrees of N-glycosylation under abnormal physiological conditions. But the key sites responsible for controlling its function remain to be determined in the context of cancer.

In conclusion, a novel technique process was successfully established for characterization of *de novo* synthesized proteins and glycoproteins during glycosylation inhibition, which would promote the clinical application of NGI-1 in future cancer treatment. We also proposed that NGI-1 induced lysosomal defects probably by impairing the lysosomal membrane protein LAMP2.

## Data Availability

The datasets presented in this study can be found in online repositories. The names of the repository/repositories and accession number(s) can be found in the article/Supplementary Material.

## Glossary

OST: Oligosaccharyltransferase
UPR: Unfolded protein response
PAGE: polyacrylamide gel electrophoresis
EGFR: Epidermal growth factor receptor
F: Fucose
BONCAT: Bioorthogonal non-canonical amino-acid tagging
LAMP2: Lysosome-associated membrane protein-2
DMEM: Dulbecco’s modified Eagle’s medium
FBS: Fetal bovine serum
N: N-acetylhexosamine
NGI-1: N-linked glycosylation inhibitor-1
AHA: Azidohomoalanine
DTT: Dithiothreitol
H: Hexose
HPLC: High-pressure liquid chromatography
GO: Gene ontology
CC: Cellular component
MF: Molecular function
BP: Biological process
S: Sialic acid
SDS: Sodium dodecyl sulfate
siRNA: Small interfering RNA
CCK8: Cell Counting Kit-8
FC: Fold change
KEGG: Kyoto Encyclopedia of Genes and Genomes
HCC: Hepatocellular carcinoma
IAA: Iodoacetamide
LC-MS/MS: Liquid chromatography-tandem mass spectrometry
PBS: Phosphate buffered saline
ER: Endoplasmic reticulum
HILIC: Hydrophilic interaction liquid chromatography.
