# Supplementary material for: Nascent Glycoproteome Reveals That N-Linked Glycosylation Inhibitor-1 Suppresses Expression of Glycosylated Lysosome-Associated Membrane Protein-2
Source: Front Mol Biosci. 2022 Apr 27;9:899192. doi: 10.3389/fmolb.2022.899192 (PMC9092021; doi:10.3389/fmolb.2022.899192)
Supplement: Supplementary file 1 [file DataSheet1.doc]

**Supplementary material**

**Figure S1.** Representative western blot for LAMP2 protein in Huh7 cells.

**
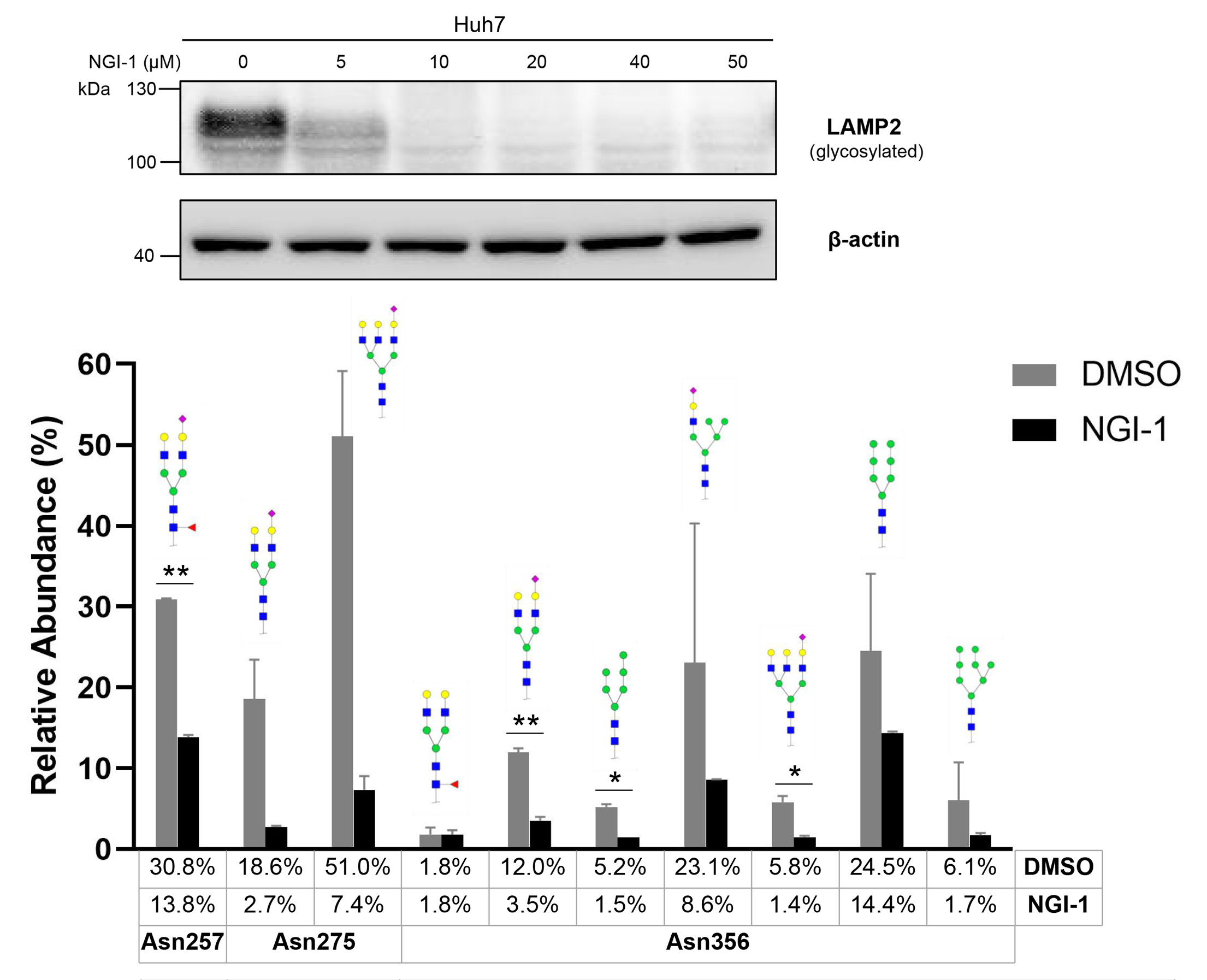
**

**Table S1. The 78 de novo proteins with a statistically significant change during NGI-1 treatment**

| **Protein accession number** | **Protein** | **Mean ratio (NGI trm/DMSO)** | **P-value** | **Alterations in NGI-1 treatment** ^△^ |
| --- | --- | --- | --- | --- |
| O75354 | ENTP6 | 0.37 | 0.0000 | ↓ |
| O00425 | IF2B3 | 3.94 | 0.0000 | ↑ |
| O94855 | SC24D | 2.17 | 0.0003 | ↑ |
| Q9NQW6 | ANLN | 2.08 | 0.0004 | ↑ |
| P11021 | BIP | 2.69 | 0.0007 | ↑ |
| Q15006 | EMC2 | 2.52 | 0.0009 | ↑ |
| Q14151 | SAFB2 | 0.39 | 0.0013 | ↓ |
| Q9BRZ2 | TRI56 | 3.30 | 0.0016 | ↑ |
| Q8NE01 | CNNM3 | 0.48 | 0.0016 | ↓ |
| Q9H6F5 | CCD86 | 0.40 | 0.0019 | ↓ |
| Q8WUA2 | PPIL4 | 0.49 | 0.0020 | ↓ |
| P30566 | PUR8 | 2.27 | 0.0024 | ↑ |
| Q58FF3 | ENPLL | 2.05 | 0.0026 | ↑ |
| P0DMV8 | HS71A | 0.33 | 0.0026 | ↓ |
| P0DMV9 | HS71B | 0.33 | 0.0026 | ↓ |
| P07339 | CATD | 0.43 | 0.0028 | ↓ |
| Q9NR22 | ANM8 | 6.55 | 0.0030 | ↑ |
| O14657 | TOR1B | 0.47 | 0.0032 | ↓ |
| Q9Y6A5 | TACC3 | 2.80 | 0.0036 | ↑ |
| P50281 | MMP14 | 0.40 | 0.0038 | ↓ |
| Q9P2D0 | IBTK | 6.84 | 0.0111 | ↑ |
| P08962 | CD63 | 2.79 | 0.0112 | ↑ |
| P13473 | LAMP2 | 0.46 | 0.0117 | ↓ |
| Q8NC56 | LEMD2 | 0.30 | 0.0121 | ↓ |
| Q5JU85 | IQEC2 | 2.05 | 0.0121 | ↑ |
| Q6DN90 | IQEC1 | 2.05 | 0.0121 | ↑ |
| Q96SL1 | DIRC2 | 0.45 | 0.0124 | ↓ |
| Q3ZCM7 | TBB8 | 2.09 | 0.0128 | ↑ |
| O75787 | RENR | 0.47 | 0.0133 | ↓ |
| Q9GZZ1 | NAA50 | 0.42 | 0.0146 | ↓ |
| P55058 | PLTP | 0.32 | 0.0148 | ↓ |
| Q9P0U4 | CXXC1 | 4.23 | 0.0155 | ↑ |
| P17066 | HSP76 | 2.59 | 0.0157 | ↑ |
| Q01105 | SET | 0.37 | 0.0159 | ↓ |
| O75592 | MYCB2 | 2.04 | 0.0160 | ↑ |
| Q9BV68 | RN126 | 0.35 | 0.0167 | ↓ |
| Q9UBW7 | ZMYM2 | 0.44 | 0.0173 | ↓ |
| P82675 | RT05 | 2.46 | 0.0173 | ↑ |
| Q9NTX5 | ECHD1 | 0.35 | 0.0192 | ↓ |
| Q9NP61 | ARFG3 | 2.57 | 0.0196 | ↑ |
| Q9NQS3 | NECT3 | 0.49 | 0.0197 | ↓ |
| Q5THJ4 | VP13D | 0.40 | 0.0198 | ↓ |
| P21108 | PRPS3 | 2.21 | 0.0208 | ↑ |
| Q5T3I0 | GPTC4 | 0.47 | 0.0215 | ↓ |
| P11234 | RALB | 0.45 | 0.0221 | ↓ |
| Q96BY9 | SARAF | 0.36 | 0.0222 | ↓ |
| Q93075 | TATD2 | 0.46 | 0.0248 | ↓ |
| Q15011 | HERP1 | 3.31 | 0.0257 | ↑ |
| Q96NB2 | SFXN2 | 0.41 | 0.0269 | ↓ |
| P17029 | ZKSC1 | 3.13 | 0.0270 | ↑ |
| O14745 | NHRF1 | 0.48 | 0.0274 | ↓ |
| Q96SK2 | TM209 | 2.47 | 0.0275 | ↑ |
| Q9H0L4 | CSTFT | 0.47 | 0.0275 | ↓ |
| Q5UCC4 | EMC10 | 0.33 | 0.0276 | ↓ |
| Q99519 | NEUR1 | 0.33 | 0.0278 | ↓ |
| Q7LGC8 | CHST3 | 0.25 | 0.0281 | ↓ |
| Q5T4D3 | TMTC4 | 0.43 | 0.0290 | ↓ |
| O95858 | TSN15 | 0.44 | 0.0292 | ↓ |
| O00192 | ARVC | 0.49 | 0.0298 | ↓ |
| Q96HP0 | DOCK6 | 2.58 | 0.0300 | ↑ |
| Q9UPP2 | IQEC3 | 2.12 | 0.0322 | ↑ |
| Q13232 | NDK3 | 0.36 | 0.0340 | ↓ |
| Q99757 | THIOM | 0.45 | 0.0347 | ↓ |
| P32456 | GBP2 | 0.37 | 0.0363 | ↓ |
| Q7Z6J4 | FGD2 | 0.26 | 0.0367 | ↓ |
| P35240 | MERL | 0.47 | 0.0370 | ↓ |
| Q96J92 | WNK4 | 0.33 | 0.0387 | ↓ |
| O43929 | ORC4 | 0.34 | 0.0390 | ↓ |
| Q8TD19 | NEK9 | 2.44 | 0.0417 | ↑ |
| Q969G5 | CAVN3 | 2.75 | 0.0420 | ↑ |
| P07858 | CATB | 0.43 | 0.0434 | ↓ |
| P0DME0 | SETLP | 0.26 | 0.0455 | ↓ |
| P14543 | NID1 | 0.31 | 0.0463 | ↓ |
| O75531 | BAF | 0.34 | 0.0464 | ↓ |
| Q8TE82 | S3TC1 | 2.45 | 0.0468 | ↑ |
| P78332 | RBM6 | 0.40 | 0.0481 | ↓ |
| P20073 | ANXA7 | 2.13 | 0.0486 | ↑ |
| P62081 | RS7 | 2.01 | 0.0499 | ↑ |

**Table S2. The identified 436 N-glycopeptides of newly synthesized proteins in NGI-1 treatment**

| **Protein accession number** | **Protein** | **N-glycosylation Sites** | **Glycan (H N S F)** | **Glycan Compositions** |
| --- | --- | --- | --- | --- |
| O00469 | PLOD2 | 209 | 9 2 0 0 | H9N2 |
| O14524 | NEMP1 | 125 | 8 2 0 0 | H8N2 |
| O14657 | TOR1B | 64 | 8 2 0 0 | H8N2 |
| O14657 | TOR1B | 64 | 9 2 0 0 | H9N2 |
| O14672 | ADA10 | 278 | 8 2 0 0 | H8N2 |
| O14672 | ADA10 | 278 | 9 2 0 0 | H9N2 |
| O14672 | ADA10 | 439 | 6 2 0 0 | H6N2 |
| O14672 | ADA10 | 439 | 7 2 0 0 | H7N2 |
| O14672 | ADA10 | 439 | 8 2 0 0 | H8N2 |
| O14672 | ADA10 | 439 | 9 2 0 0 | H9N2 |
| O60568 | PLOD3 | 63 | 6 2 0 0 | H6N2 |
| O60568 | PLOD3 | 548 | 6 2 0 0 | H6N2 |
| O60568 | PLOD3 | 548 | 8 2 0 0 | H8N2 |
| O60568 | PLOD3 | 548 | 9 2 0 0 | H9N2 |
| O60637 | TSN3 | 167 | 5 2 0 0 | H5N2 |
| O60637 | TSN3 | 167 | 5 3 1 1 | H5N3S1F1 |
| O60637 | TSN3 | 167 | 6 2 0 0 | H6N2 |
| O60637 | TSN3 | 167 | 6 3 1 0 | H6N3S1 |
| O60637 | TSN3 | 167 | 7 2 0 0 | H7N2 |
| O75477/O94905 | ERLN1/ERLN2 | 108/106 | 7 2 0 0 | H7N2 |
| O75477/O94905 | ERLN1/ERLN2 | 108/106 | 8 2 0 0 | H8N2 |
| O75477/O94905 | ERLN1/ERLN2 | 108/106 | 9 2 0 0 | H9N2 |
| O75976 | CBPD | 626 | 5 3 1 0 | H5N3S1 |
| O75976 | CBPD | 626 | 6 3 1 0 | H6N3S1 |
| O75976 | CBPD | 626 | 8 2 0 0 | H8N2 |
| O94901 | SUN1 | 588 | 8 2 0 0 | H8N2 |
| O94901 | SUN1 | 732 | 6 2 0 0 | H6N2 |
| O95302 | FKBP9 | 286 | 8 2 0 0 | H8N2 |
| O95302 | FKBP9 | 286 | 9 2 0 0 | H9N2 |
| O96005 | CLPT1 | 295 | 8 2 0 0 | H8N2 |
| P00450 | CERU | 138 | 7 2 0 0 | H7N2 |
| P00533 | EGFR | 352 | 6 2 0 0 | H6N2 |
| P00533 | EGFR | 352 | 7 2 0 0 | H7N2 |
| P00533 | EGFR | 352 | 8 2 0 0 | H8N2 |
| P00533 | EGFR | 352 | 9 2 0 0 | H9N2 |
| P00739/P00738 | HPTR/HPT | 126/184 | 5 4 1 0 | H5N4S1 |
| P00739/P00738 | HPTR/HPT | 126/184 | 5 4 2 0 | H5N4S2 |
| P02679 | FIBG | 78 | 5 4 0 0 | H5N4 |
| P02679 | FIBG | 78 | 5 4 1 0 | H5N4S1 |
| P02679 | FIBG | 78 | 5 4 1 1 | H5N4S1F1 |
| P02679 | FIBG | 78 | 5 4 2 0 | H5N4S2 |
| P02679 | FIBG | 78 | 5 5 1 1 | H5N5S1F1 |
| P02679 | FIBG | 78 | 8 2 0 0 | H8N2 |
| P02751 | FINC | 528 | 5 4 1 1 | H5N4S1F1 |
| P02751 | FINC | 528 | 8 2 0 0 | H8N2 |
| P02751 | FINC | 528 | 9 2 0 0 | H9N2 |
| P02751 | FINC | 1007 | 6 4 0 2 | H6N4F2 |
| P02751 | FINC | 1007 | 6 4 1 2 | H6N4S1F2 |
| P02771 | FETA | 251 | 3 4 0 1 | H3N4F1 |
| P02771 | FETA | 251 | 4 3 0 1 | H4N3F1 |
| P02771 | FETA | 251 | 5 4 1 1 | H5N4S1F1 |
| P02771 | FETA | 251 | 5 4 2 1 | H5N4S2F1 |
| P02771 | FETA | 251 | 5 5 2 0 | H5N5S2 |
| P02771 | FETA | 251 | 7 2 0 0 | H7N2 |
| P02771 | FETA | 251 | 8 2 0 0 | H8N2 |
| P02786 | TFR1 | 727 | 7 2 0 0 | H7N2 |
| P02786 | TFR1 | 727 | 8 2 0 0 | H8N2 |
| P02786 | TFR1 | 727 | 9 2 0 0 | H9N2 |
| P04004 | VTNC | 86 | 8 2 0 0 | H8N2 |
| P04004 | VTNC | 169 | 9 2 0 0 | H9N2 |
| P04062 | GLCM | 309 | 3 2 0 1 | H3N2F1 |
| P04843 | RPN1 | 299 | 7 2 0 0 | H7N2 |
| P04843 | RPN1 | 299 | 8 2 0 0 | H8N2 |
| P05556 | ITB1 | 212 | 4 4 1 2 | H4N4S1F2 |
| P05556 | ITB1 | 212 | 7 2 0 0 | H7N2 |
| P05556 | ITB1 | 212 | 8 2 0 0 | H8N2 |
| P05556 | ITB1 | 212 | 9 2 0 0 | H9N2 |
| P05556 | ITB1 | 481 | 6 2 0 0 | H6N2 |
| P05556 | ITB1 | 481 | 7 2 0 0 | H7N2 |
| P05556 | ITB1 | 481 | 8 2 0 0 | H8N2 |
| P05556 | ITB1 | 481 | 9 2 0 0 | H9N2 |
| P05556 | ITB1 | 520 | 7 2 0 0 | H7N2 |
| P05556 | ITB1 | 520 | 8 2 0 0 | H8N2 |
| P05556 | ITB1 | 669 | 5 3 1 0 | H5N3S1 |
| P05556 | ITB1 | 669 | 6 2 0 0 | H6N2 |
| P05556 | ITB1 | 669 | 7 2 0 0 | H7N2 |
| P05556 | ITB1 | 669 | 8 2 0 0 | H8N2 |
| P05556 | ITB1 | 669 | 9 2 0 0 | H9N2 |
| P06213 | INSR | 445 | 9 2 0 0 | H9N2 |
| P06756 | ITAV | 74 | 8 2 0 0 | H8N2 |
| P06756 | ITAV | 615 | 9 2 0 0 | H9N2 |
| P06756 | ITAV | 874 | 5 2 0 0 | H5N2 |
| P06756 | ITAV | 874 | 6 2 0 0 | H6N2 |
| P06756 | ITAV | 874 | 7 2 0 0 | H7N2 |
| P06756 | ITAV | 874 | 8 2 0 0 | H8N2 |
| P06865 | HEXA | 157 | 8 2 0 0 | H8N2 |
| P07339 | CATD | 263 | 6 2 0 0 | H6N2 |
| P07602 | SAP | 215 | 3 2 0 1 | H3N2F1 |
| P07602 | SAP | 215 | 5 4 1 1 | H5N4S1F1 |
| P07602 | SAP | 215 | 5 4 2 1 | H5N4S2F1 |
| P07602 | SAP | 215 | 8 2 0 0 | H8N2 |
| P07602 | SAP | 215 | 9 2 0 0 | H9N2 |
| P07602 | SAP | 332 | 3 2 0 1 | H3N2F1 |
| P07602 | SAP | 332 | 4 2 0 0 | H4N2 |
| P07602 | SAP | 332 | 5 2 0 0 | H5N2 |
| P07602 | SAP | 332 | 6 2 0 0 | H6N2 |
| P07711 | CATL1 | 221 | 6 3 2 0 | H6N3S2 |
| P07942 | LAMB1 | 677 | 6 2 0 0 | H6N2 |
| P07942 | LAMB1 | 677 | 8 2 0 0 | H8N2 |
| P07942 | LAMB1 | 677 | 9 2 0 0 | H9N2 |
| P07942 | LAMB1 | 1279 | 6 2 0 0 | H6N2 |
| P07942 | LAMB1 | 1279 | 8 2 0 0 | H8N2 |
| P08581 | MET | 106 | 7 2 0 0 | H7N2 |
| P08581 | MET | 106 | 8 2 0 0 | H8N2 |
| P08581 | MET | 106 | 9 2 0 0 | H9N2 |
| P08581 | MET | 785 | 10 2 0 0 | H10N2 |
| P08581 | MET | 785 | 5 2 0 0 | H5N2 |
| P08581 | MET | 785 | 5 4 1 0 | H5N4S1 |
| P08581 | MET | 785 | 5 4 1 1 | H5N4S1F1 |
| P08581 | MET | 785 | 5 4 2 1 | H5N4S2F1 |
| P08581 | MET | 785 | 5 5 0 2 | H5N5F2 |
| P08581 | MET | 785 | 6 2 0 0 | H6N2 |
| P08581 | MET | 785 | 6 5 1 1 | H6N5S1F1 |
| P08581 | MET | 785 | 7 2 0 0 | H7N2 |
| P08581 | MET | 785 | 8 2 0 0 | H8N2 |
| P08581 | MET | 785 | 9 2 0 0 | H9N2 |
| P10316/P01892 | 1A69/1A02 | 110/110 | 8 2 0 0 | H8N2 |
| P10909 | CLUS | 86 | 6 2 0 0 | H6N2 |
| P10909 | CLUS | 86 | 7 2 0 0 | H7N2 |
| P10909 | CLUS | 86 | 8 2 0 0 | H8N2 |
| P10909 | CLUS | 86 | 9 2 0 0 | H9N2 |
| P10909 | CLUS | 291 | 5 4 2 1 | H5N4S2F1 |
| P10909 | CLUS | 374 | 6 2 0 0 | H6N2 |
| P10909 | CLUS | 374 | 7 2 0 0 | H7N2 |
| P10909 | CLUS | 374 | 8 2 0 0 | H8N2 |
| P11047 | LAMC1 | 650 | 8 2 0 0 | H8N2 |
| P11047 | LAMC1 | 650 | 9 2 0 0 | H9N2 |
| P11047 | LAMC1 | 1107 | 6 2 0 0 | H6N2 |
| P11047 | LAMC1 | 1107 | 8 2 0 0 | H8N2 |
| P11047 | LAMC1 | 1241 | 8 2 0 0 | H8N2 |
| P11047 | LAMC1 | 1395 | 5 2 0 0 | H5N2 |
| P11047 | LAMC1 | 1395 | 6 2 0 0 | H6N2 |
| P11047 | LAMC1 | 1395 | 7 2 0 0 | H7N2 |
| P11047 | LAMC1 | 1395 | 8 2 0 0 | H8N2 |
| P11117 | PPAL | 331 | 6 2 0 0 | H6N2 |
| P11279 | LAMP1 | 84 | 8 6 1 2 | H8N6S1F2 |
| P11279 | LAMP1 | 103 | 5 3 1 0 | H5N3S1 |
| P11279 | LAMP1 | 103 | 6 2 0 0 | H6N2 |
| P11279 | LAMP1 | 103 | 6 3 1 0 | H6N3S1 |
| P11279 | LAMP1 | 103 | 7 2 0 0 | H7N2 |
| P11279 | LAMP1 | 249 | 5 4 2 1 | H5N4S2F1 |
| P11279 | LAMP1 | 249 | 6 5 2 1 | H6N5S2F1 |
| P11279 | LAMP1 | 249 | 6 5 3 1 | H6N5S3F1 |
| P11279 | LAMP1 | 261 | 7 2 0 0 | H7N2 |
| P11717 | MPRI | 112 | 6 2 0 0 | H6N2 |
| P11717 | MPRI | 112 | 8 2 0 0 | H8N2 |
| P11717 | MPRI | 112 | 9 2 0 0 | H9N2 |
| P11717 | MPRI | 400 | 8 2 0 0 | H8N2 |
| P11717 | MPRI | 581 | 7 2 0 0 | H7N2 |
| P11717 | MPRI | 581 | 8 2 0 0 | H8N2 |
| P11717 | MPRI | 581 | 9 2 0 0 | H9N2 |
| P11717 | MPRI | 1656 | 5 4 2 1 | H5N4S2F1 |
| P11717 | MPRI | 1656 | 8 2 0 0 | H8N2 |
| P11717 | MPRI | 1656 | 9 2 0 0 | H9N2 |
| P12109 | CO6A1 | 212 | 8 2 0 0 | H8N2 |
| P12109 | CO6A1 | 804 | 8 2 0 0 | H8N2 |
| P13473 | LAMP2 | 257 | 5 4 1 1 | H5N4S1F1 |
| P13473 | LAMP2 | 257 | 8 2 0 0 | H8N2 |
| P13473 | LAMP2 | 257 | 9 2 0 0 | H9N2 |
| P13473 | LAMP2 | 275 | 5 4 1 0 | H5N4S1 |
| P13473 | LAMP2 | 275 | 6 5 1 0 | H6N5S1 |
| P13473 | LAMP2 | 275 | 7 6 1 0 | H7N6S1 |
| P13473 | LAMP2 | 275 | 8 6 0 1 | H8N6F1 |
| P13473 | LAMP2 | 275 | 9 7 0 1 | H9N7F1 |
| P13473 | LAMP2 | 356 | 4 3 1 0 | H4N3S1 |
| P13473 | LAMP2 | 356 | 5 2 0 0 | H5N2 |
| P13473 | LAMP2 | 356 | 5 3 1 0 | H5N3S1 |
| P13473 | LAMP2 | 356 | 5 4 1 0 | H5N4S1 |
| P13473 | LAMP2 | 356 | 5 4 1 1 | H5N4S1F1 |
| P13473 | LAMP2 | 356 | 5 4 2 1 | H5N4S2F1 |
| P13473 | LAMP2 | 356 | 6 2 0 0 | H6N2 |
| P13473 | LAMP2 | 356 | 6 3 1 0 | H6N3S1 |
| P13473 | LAMP2 | 356 | 6 5 1 1 | H6N5S1F1 |
| P13473 | LAMP2 | 356 | 7 2 0 0 | H7N2 |
| P13473 | LAMP2 | 356 | 7 5 0 2 | H7N5F2 |
| P13473 | LAMP2 | 356 | 7 5 1 1 | H7N5S1F1 |
| P13473 | LAMP2 | 356 | 8 2 0 0 | H8N2 |
| P13473 | LAMP2 | 356 | 9 2 0 0 | H9N2 |
| P13688 | CEAM1 | 378 | 8 2 0 0 | H8N2 |
| P14625 | ENPL | 217 | 3 2 0 1 | H3N2F1 |
| P14625 | ENPL | 217 | 3 3 0 0 | H3N3 |
| P14625 | ENPL | 217 | 3 3 0 1 | H3N3F1 |
| P14625 | ENPL | 217 | 3 4 0 1 | H3N4F1 |
| P14625 | ENPL | 217 | 5 2 0 0 | H5N2 |
| P14625 | ENPL | 217 | 6 2 0 0 | H6N2 |
| P14625 | ENPL | 217 | 7 2 0 0 | H7N2 |
| P14625 | ENPL | 217 | 8 2 0 0 | H8N2 |
| P14625 | ENPL | 217 | 9 2 0 0 | H9N2 |
| P14625 | ENPL | 445 | 8 2 0 0 | H8N2 |
| P14625 | ENPL | 481 | 8 2 0 0 | H8N2 |
| P14625 | ENPL | 481 | 9 2 0 0 | H9N2 |
| P15144 | AMPN | 128 | 5 2 0 0 | H5N2 |
| P15144 | AMPN | 128 | 6 2 0 0 | H6N2 |
| P15144 | AMPN | 128 | 7 2 0 0 | H7N2 |
| P15144 | AMPN | 128 | 8 2 0 0 | H8N2 |
| P15586 | GNS | 362 | 4 7 0 0 | H4N7 |
| P15586 | GNS | 362 | 6 2 0 0 | H6N2 |
| P15586 | GNS | 362 | 7 2 0 0 | H7N2 |
| P15586 | GNS | 362 | 8 2 0 0 | H8N2 |
| P15586 | GNS | 362 | 9 2 0 0 | H9N2 |
| P19022 | CADH2 | 651 | 8 2 0 0 | H8N2 |
| P19022 | CADH2 | 651 | 9 2 0 0 | H9N2 |
| P20645 | MPRD | 57 | 6 2 0 0 | H6N2 |
| P20645 | MPRD | 57 | 7 2 0 0 | H7N2 |
| P20645 | MPRD | 57 | 8 2 0 0 | H8N2 |
| P20645 | MPRD | 83 | 8 2 0 0 | H8N2 |
| P21589 | 5NTD | 311 | 7 2 0 0 | H7N2 |
| P23229 | ITA6 | 323 | 8 2 0 0 | H8N2 |
| P26006 | ITA3 | 265 | 9 2 0 0 | H9N2 |
| P35613 | BASI | 160 | 6 2 0 0 | H6N2 |
| P35613 | BASI | 160 | 8 2 0 0 | H8N2 |
| P40199 | CEAM6 | 224 | 3 2 0 0 | H3N2 |
| P40199 | CEAM6 | 224 | 6 2 0 0 | H6N2 |
| P42702 | LIFR | 787 | 9 2 0 0 | H9N2 |
| P42892 | ECE1 | 166 | 8 2 0 0 | H8N2 |
| P42892 | ECE1 | 166 | 9 2 0 0 | H9N2 |
| P42892 | ECE1 | 270 | 9 2 0 0 | H9N2 |
| P43308 | SSRB | 88 | 10 2 0 0 | H10N2 |
| P43308 | SSRB | 88 | 7 2 0 0 | H7N2 |
| P43308 | SSRB | 88 | 8 2 0 0 | H8N2 |
| P43308 | SSRB | 88 | 9 2 0 0 | H9N2 |
| P46977 | STT3A | 544 | 7 2 0 0 | H7N2 |
| P46977 | STT3A | 544 | 8 2 0 0 | H8N2 |
| P46977 | STT3A | 544 | 9 2 0 0 | H9N2 |
| P46977 | STT3A | 548 | 10 2 0 0 | H10N2 |
| P46977 | STT3A | 548 | 6 2 0 0 | H6N2 |
| P46977 | STT3A | 548 | 7 2 0 0 | H7N2 |
| P46977 | STT3A | 548 | 8 2 0 0 | H8N2 |
| P46977 | STT3A | 548 | 9 2 0 0 | H9N2 |
| P48723 | HSP13 | 184 | 8 2 0 0 | H8N2 |
| P51690 | ARSE | 125 | 8 2 0 0 | H8N2 |
| P51690 | ARSE | 258 | 8 2 0 0 | H8N2 |
| P51690 | ARSE | 258 | 9 2 0 0 | H9N2 |
| P53634 | CATC | 119 | 7 2 0 0 | H7N2 |
| P53801 | PTTG | 54 | 6 2 0 0 | H6N2 |
| P53801 | PTTG | 54 | 7 2 0 0 | H7N2 |
| P54709 | AT1B3 | 124 | 8 2 0 0 | H8N2 |
| P55268 | LAMB2 | 1348 | 8 2 0 0 | H8N2 |
| P55283/P19022 | CADH4/CADH2 | 661/651 | 8 2 0 0 | H8N2 |
| P56199 | ITA1 | 883 | 9 2 0 0 | H9N2 |
| P69849/Q15155/Q5JPE7 | NOMO3/NOMO1/NOMO2 | 369/369/369 | 6 2 0 0 | H6N2 |
| P69849/Q15155/Q5JPE7 | NOMO3/NOMO1/NOMO2 | 369/369/369 | 7 2 0 0 | H7N2 |
| P69849/Q15155/Q5JPE7 | NOMO3/NOMO1/NOMO2 | 369/369/369 | 8 2 0 0 | H8N2 |
| P69849/Q15155/Q5JPE7 | NOMO3/NOMO1/NOMO2 | 369/369/369 | 9 2 0 0 | H9N2 |
| P69849/Q15155/Q5JPE7 | NOMO3/NOMO1/NOMO2 | 618/618/618 | 7 2 0 0 | H7N2 |
| P69849/Q15155/Q5JPE7 | NOMO3/NOMO1/NOMO2 | 618/618/618 | 8 2 0 0 | H8N2 |
| P69849/Q15155/Q5JPE7 | NOMO3/NOMO1/NOMO2 | 618/618/618 | 9 2 0 0 | H9N2 |
| P78536 | ADA17 | 264 | 7 2 0 0 | H7N2 |
| P78536 | ADA17 | 539 | 8 2 0 0 | H8N2 |
| P78536 | ADA17 | 594 | 8 2 0 0 | H8N2 |
| Q02809 | PLOD1 | 538 | 8 2 0 0 | H8N2 |
| Q02809 | PLOD1 | 538 | 9 2 0 0 | H9N2 |
| Q07954 | LRP1 | 446 | 8 2 0 0 | H8N2 |
| Q07954 | LRP1 | 2127 | 8 2 0 0 | H8N2 |
| Q07954 | LRP1 | 2127 | 9 2 0 0 | H9N2 |
| Q07954 | LRP1 | 2620 | 8 2 0 0 | H8N2 |
| Q07954 | LRP1 | 3333 | 9 2 0 0 | H9N2 |
| Q07954 | LRP1 | 4075 | 4 4 1 2 | H4N4S1F2 |
| Q07954 | LRP1 | 4075 | 7 2 0 0 | H7N2 |
| Q07954 | LRP1 | 4075 | 8 2 0 0 | H8N2 |
| Q07954 | LRP1 | 4075 | 9 2 0 0 | H9N2 |
| Q07954 | LRP1 | 4125 | 8 2 0 0 | H8N2 |
| Q07954 | LRP1 | 4179 | 9 2 0 0 | H9N2 |
| Q07954 | LRP1 | 4364 | 8 2 0 0 | H8N2 |
| Q07954/Q9NZR2 | LRP1/LRP1B | 1995/1983 | 6 2 0 0 | H6N2 |
| Q08380 | LG3BP | 69 | 8 2 0 0 | H8N2 |
| Q08380 | LG3BP | 125 | 6 2 0 0 | H6N2 |
| Q08380 | LG3BP | 125 | 8 2 0 0 | H8N2 |
| Q08380 | LG3BP | 125 | 9 2 0 0 | H9N2 |
| Q08380 | LG3BP | 398 | 6 2 0 0 | H6N2 |
| Q08380 | LG3BP | 398 | 7 2 0 0 | H7N2 |
| Q08380 | LG3BP | 398 | 8 2 0 0 | H8N2 |
| Q08380 | LG3BP | 398 | 9 2 0 0 | H9N2 |
| Q08380 | LG3BP | 551 | 6 2 0 0 | H6N2 |
| Q08380 | LG3BP | 551 | 7 2 0 0 | H7N2 |
| Q08380 | LG3BP | 551 | 8 2 0 0 | H8N2 |
| Q08380 | LG3BP | 551 | 9 2 0 0 | H9N2 |
| Q12797 | ASPH | 452 | 5 2 0 0 | H5N2 |
| Q12797 | ASPH | 452 | 6 2 0 0 | H6N2 |
| Q12797 | ASPH | 452 | 7 2 0 0 | H7N2 |
| Q12797 | ASPH | 452 | 8 2 0 0 | H8N2 |
| Q12797 | ASPH | 452 | 9 2 0 0 | H9N2 |
| Q13443 | ADAM9 | 381 | 7 2 0 0 | H7N2 |
| Q13443 | ADAM9 | 381 | 8 2 0 0 | H8N2 |
| Q13510 | ASAH1 | 259 | 6 2 0 0 | H6N2 |
| Q13510 | ASAH1 | 259 | 7 2 0 0 | H7N2 |
| Q13510 | ASAH1 | 259 | 8 2 0 0 | H8N2 |
| Q13641 | TPBG | 124 | 7 2 0 0 | H7N2 |
| Q13641 | TPBG | 124 | 8 2 0 0 | H8N2 |
| Q13740 | CD166 | 306 | 5 4 2 1 | H5N4S2F1 |
| Q14108 | SCRB2 | 105 | 8 2 0 0 | H8N2 |
| Q15165 | PON2 | 254 | 8 2 0 0 | H8N2 |
| Q15904 | VAS1 | 261 | 8 2 0 0 | H8N2 |
| Q16563 | SYPL1 | 71 | 6 3 1 0 | H6N3S1 |
| Q16563 | SYPL1 | 71 | 7 3 0 1 | H7N3F1 |
| Q32NC0 | CR021 | 108 | 6 2 0 0 | H6N2 |
| Q32P28 | P3H1 | 467 | 6 2 0 0 | H6N2 |
| Q32P28 | P3H1 | 467 | 7 2 0 0 | H7N2 |
| Q32P28 | P3H1 | 540 | 8 2 0 0 | H8N2 |
| Q4KMQ2 | ANO6 | 361 | 7 2 0 0 | H7N2 |
| Q4KMQ2 | ANO6 | 361 | 8 2 0 0 | H8N2 |
| Q58FF3/P14625 | ENPLL/ENPL | 159/502 | 7 2 0 0 | H7N2 |
| Q58FF3/P14625 | ENPLL/ENPL | 159/502 | 8 2 0 0 | H8N2 |
| Q58FF3/P14625 | ENPLL/ENPL | 159/502 | 9 2 0 0 | H9N2 |
| Q5JRA6 | TGO1 | 246 | 6 2 0 0 | H6N2 |
| Q5JRA6 | TGO1 | 246 | 7 2 0 0 | H7N2 |
| Q5JRA6 | TGO1 | 246 | 8 2 0 0 | H8N2 |
| Q5JRA6 | TGO1 | 250 | 5 2 0 0 | H5N2 |
| Q5JRA6 | TGO1 | 250 | 6 2 0 0 | H6N2 |
| Q5JRA6 | TGO1 | 250 | 7 2 0 0 | H7N2 |
| Q5JRA6 | TGO1 | 250 | 8 2 0 0 | H8N2 |
| Q6P4E1 | CASC4 | 150 | 5 4 2 1 | H5N4S2F1 |
| Q6UXH1 | CREL2 | 190 | 6 2 0 0 | H6N2 |
| Q6UXH1 | CREL2 | 190 | 8 2 0 0 | H8N2 |
| Q6YHK3 | CD109 | 337 | 8 2 0 0 | H8N2 |
| Q6YHK3 | CD109 | 337 | 9 2 0 0 | H9N2 |
| Q6ZNA5 | FRRS1 | 308 | 9 2 0 0 | H9N2 |
| Q7LGA3 | HS2ST | 108 | 6 3 1 0 | H6N3S1 |
| Q7Z388 | D19L4 | 123 | 6 2 0 0 | H6N2 |
| Q86SQ4 | AGRG6 | 750 | 8 2 0 0 | H8N2 |
| Q8IV08 | PLD3 | 387 | 8 2 0 0 | H8N2 |
| Q8N766 | EMC1 | 913 | 8 2 0 0 | H8N2 |
| Q8N766 | EMC1 | 913 | 9 2 0 0 | H9N2 |
| Q8NBJ4 | GOLM1 | 109 | 5 4 2 1 | H5N4S2F1 |
| Q8NBJ4 | GOLM1 | 144 | 5 4 2 1 | H5N4S2F1 |
| Q8NBJ4 | GOLM1 | 144 | 6 2 0 0 | H6N2 |
| Q8NBJ4 | GOLM1 | 144 | 7 2 0 0 | H7N2 |
| Q8NBJ4 | GOLM1 | 144 | 8 2 0 0 | H8N2 |
| Q8NBJ4 | GOLM1 | 144 | 9 2 0 0 | H9N2 |
| Q8NFQ8 | TOIP2 | 286 | 8 2 0 0 | H8N2 |
| Q8NFQ8 | TOIP2 | 286 | 9 2 0 0 | H9N2 |
| Q8TCJ2 | STT3B | 623 | 10 2 0 0 | H10N2 |
| Q8TCJ2 | STT3B | 623 | 7 2 0 0 | H7N2 |
| Q8TCJ2 | STT3B | 623 | 8 2 0 0 | H8N2 |
| Q8TCJ2 | STT3B | 623 | 9 2 0 0 | H9N2 |
| Q8TCJ2 | STT3B | 627 | 7 2 0 0 | H7N2 |
| Q8TCJ2 | STT3B | 627 | 8 2 0 0 | H8N2 |
| Q8TCJ2 | STT3B | 627 | 9 2 0 0 | H9N2 |
| Q8TCJ2 | STT3B | 641 | 9 2 0 0 | H9N2 |
| Q8TEM1 | PO210 | 1441 | 6 2 0 0 | H6N2 |
| Q8TEM1 | PO210 | 1441 | 7 2 0 0 | H7N2 |
| Q8TEM1 | PO210 | 1441 | 8 2 0 0 | H8N2 |
| Q8WWI5 | CTL1 | 135 | 7 2 0 0 | H7N2 |
| Q92542 | NICA | 45 | 8 2 0 0 | H8N2 |
| Q92542 | NICA | 45 | 9 2 0 0 | H9N2 |
| Q92542 | NICA | 417 | 8 2 0 0 | H8N2 |
| Q92542 | NICA | 435 | 7 2 0 0 | H7N2 |
| Q92542 | NICA | 435 | 8 2 0 0 | H8N2 |
| Q969V3 | NCLN | 428 | 8 2 0 0 | H8N2 |
| Q96AY3 | FKB10 | 70 | 8 2 0 0 | H8N2 |
| Q96AY3 | FKB10 | 182 | 8 2 0 0 | H8N2 |
| Q96FV9 | THOC1 | 488 | 8 2 0 0 | H8N2 |
| Q96G97 | BSCL2 | 88 | 3 4 2 0 | H3N4S2 |
| Q96J42 | TXD15 | 187 | 10 6 1 2 | H10N6S1F2 |
| Q96KA5 | CLP1L | 91 | 5 2 0 0 | H5N2 |
| Q96KA5 | CLP1L | 91 | 6 2 0 0 | H6N2 |
| Q96KA5 | CLP1L | 91 | 7 2 0 0 | H7N2 |
| Q96KA5 | CLP1L | 91 | 8 2 0 0 | H8N2 |
| Q9BRK3 | MXRA8 | 306 | 8 2 0 0 | H8N2 |
| Q9BRN9 | TM2D3 | 169 | 8 2 0 0 | H8N2 |
| Q9BU23 | LMF2 | 616 | 7 2 0 0 | H7N2 |
| Q9BU23 | LMF2 | 616 | 8 2 0 0 | H8N2 |
| Q9BVK6 | TMED9 | 125 | 3 4 0 0 | H3N4 |
| Q9BVK6 | TMED9 | 125 | 3 4 0 1 | H3N4F1 |
| Q9BVK6 | TMED9 | 125 | 3 6 0 1 | H3N6F1 |
| Q9BVK6 | TMED9 | 125 | 5 2 0 0 | H5N2 |
| Q9BVK6 | TMED9 | 125 | 6 2 0 0 | H6N2 |
| Q9BVK6 | TMED9 | 125 | 7 2 0 0 | H7N2 |
| Q9BVK6 | TMED9 | 125 | 8 2 0 0 | H8N2 |
| Q9H330 | TM245 | 551 | 8 2 0 0 | H8N2 |
| Q9H330 | TM245 | 551 | 9 2 0 0 | H9N2 |
| Q9H3G5 | CPVL | 346 | 5 2 0 1 | H5N2F1 |
| Q9H3G5 | CPVL | 346 | 6 2 0 0 | H6N2 |
| Q9HAW8/P35504/P22309/P35503/Q9HAW7/P19224/P22310/O60656/Q9HAW9 | UD110/UD15/UD11/UD13/UD17/UD16/UD14/UD19/UD18 | 344/348/347/348/344/346/348/344/344 | 5 2 0 0 | H5N2 |
| Q9HAW8/P35504/P22309/P35503/Q9HAW7/P19224/P22310/O60656/Q9HAW9 | UD110/UD15/UD11/UD13/UD17/UD16/UD14/UD19/UD18 | 344/348/347/348/344/346/348/344/344 | 5 2 0 1 | H5N2F1 |
| Q9HAW8/P35504/P22309/P35503/Q9HAW7/P19224/P22310/O60656/Q9HAW9 | UD110/UD15/UD11/UD13/UD17/UD16/UD14/UD19/UD18 | 344/348/347/348/344/346/348/344/344 | 6 2 0 0 | H6N2 |
| Q9HAW8/P35504/P22309/P35503/Q9HAW7/P19224/P22310/O60656/Q9HAW9 | UD110/UD15/UD11/UD13/UD17/UD16/UD14/UD19/UD18 | 344/348/347/348/344/346/348/344/344 | 7 2 0 0 | H7N2 |
| Q9HAW8/P35504/P22309/P35503/Q9HAW7/P19224/P22310/O60656/Q9HAW9 | UD110/UD15/UD11/UD13/UD17/UD16/UD14/UD19/UD18 | 344/348/347/348/344/346/348/344/344 | 8 2 0 0 | H8N2 |
| Q9HAW8/P35504/P22309/P35503/Q9HAW7/P19224/P22310/O60656/Q9HAW9 | UD110/UD15/UD11/UD13/UD17/UD16/UD14/UD19/UD18 | 344/348/347/348/344/346/348/344/344 | 9 2 0 0 | H9N2 |
| Q9HAW8/Q9HAW7/O60656/Q9HAW9 | UD110/UD17/UD19/UD18 | 71/71/71/71 | 5 2 0 0 | H5N2 |
| Q9HAW8/Q9HAW7/O60656/Q9HAW9 | UD110/UD17/UD19/UD18 | 71/71/71/71 | 5 2 0 1 | H5N2F1 |
| Q9HAW8/Q9HAW7/O60656/Q9HAW9 | UD110/UD17/UD19/UD18 | 71/71/71/71 | 6 2 0 0 | H6N2 |
| Q9HAW8/Q9HAW7/O60656/Q9HAW9 | UD110/UD17/UD19/UD18 | 71/71/71/71 | 6 2 0 1 | H6N2F1 |
| Q9HAW8/Q9HAW7/O60656/Q9HAW9 | UD110/UD17/UD19/UD18 | 71/71/71/71 | 7 2 0 0 | H7N2 |
| Q9HAW8/Q9HAW7/O60656/Q9HAW9 | UD110/UD17/UD19/UD18 | 71/71/71/71 | 8 2 0 0 | H8N2 |
| Q9HD45 | TM9S3 | 174 | 5 4 2 0 | H5N4S2 |
| Q9HD45 | TM9S3 | 174 | 6 3 1 0 | H6N3S1 |
| Q9HD45 | TM9S3 | 174 | 6 4 0 2 | H6N4F2 |
| Q9HD45 | TM9S3 | 174 | 6 4 1 1 | H6N4S1S1 |
| Q9HDC9 | APMAP | 160 | 5 2 0 0 | H5N2 |
| Q9HDC9 | APMAP | 160 | 6 2 0 0 | H6N2 |
| Q9HDC9 | APMAP | 160 | 7 2 0 0 | H7N2 |
| Q9HDC9 | APMAP | 160 | 8 2 0 0 | H8N2 |
| Q9HDC9 | APMAP | 196 | 8 2 0 0 | H8N2 |
| Q9UBS4 | DJB11 | 261 | 9 2 0 0 | H9N2 |
| Q9UBS9 | SUCO | 530 | 8 2 0 0 | H8N2 |
| Q9UBV2 | SE1L1 | 608 | 8 2 0 0 | H8N2 |
| Q9UBV2 | SE1L1 | 608 | 9 2 0 0 | H9N2 |
| Q9UH99 | SUN2 | 636 | 5 2 0 0 | H5N2 |
| Q9UH99 | SUN2 | 636 | 6 2 0 0 | H6N2 |
| Q9UH99 | SUN2 | 636 | 7 2 0 0 | H7N2 |
| Q9UHG3 | PCYOX | 353 | 3 4 2 0 | H3N4S2 |
| Q9UHG3 | PCYOX | 353 | 7 2 0 0 | H7N2 |
| Q9UHG3 | PCYOX | 353 | 8 2 0 0 | H8N2 |
| Q9UHN6 | CEIP2 | 248 | 4 4 1 2 | H4N4S1F2 |
| Q9UHN6 | CEIP2 | 914 | 8 2 0 0 | H8N2 |
| Q9UHN6 | CEIP2 | 914 | 9 2 0 0 | H9N2 |
| Q9UHN6 | CEIP2 | 980 | 5 4 1 0 | H5N4S1 |
| Q9UHN6 | CEIP2 | 980 | 5 4 1 1 | H5N4S1F1 |
| Q9UHN6 | CEIP2 | 1092 | 5 4 1 1 | H5N4S1F1 |
| Q9UHN6 | CEIP2 | 1234 | 3 4 2 0 | H3N4S2 |
| Q9UJ14 | GGT7 | 198 | 8 2 0 0 | H8N2 |
| Q9ULU4 | PKCB1 | 361 | 6 2 0 0 | H6N2 |
| Q9ULW0 | TPX2 | 308 | 3 4 0 1 | H3N4F1 |
| Q9ULW0 | TPX2 | 308 | 3 5 0 0 | H3N5 |
| Q9ULW0 | TPX2 | 308 | 4 4 0 1 | H4N4F1 |
| Q9ULW0 | TPX2 | 308 | 4 6 1 0 | H4N6S1 |
| Q9ULW0 | TPX2 | 308 | 5 4 0 1 | H5N4F1 |
| Q9ULW0 | TPX2 | 308 | 5 5 0 0 | H5N5 |
| Q9ULW0 | TPX2 | 308 | 6 4 0 1 | H6N4F1 |
| Q9ULW0 | TPX2 | 308 | 7 4 0 1 | H7N4F1 |
| Q9Y4L1 | HYOU1 | 515 | 3 4 2 0 | H3N4S2 |
| Q9Y4L1 | HYOU1 | 515 | 7 2 0 0 | H7N2 |
| Q9Y4L1 | HYOU1 | 515 | 8 2 0 0 | H8N2 |
| Q9Y4L1 | HYOU1 | 515 | 9 2 0 0 | H9N2 |
| Q9Y4L1 | HYOU1 | 596 | 8 2 0 0 | H8N2 |
| Q9Y4L1 | HYOU1 | 862 | 8 2 0 0 | H8N2 |
| Q9Y4L1 | HYOU1 | 869 | 6 2 0 0 | H6N2 |
| Q9Y4L1 | HYOU1 | 869 | 7 2 0 0 | H7N2 |
| Q9Y4L1 | HYOU1 | 869 | 8 2 0 0 | H8N2 |
| Q9Y4L1 | HYOU1 | 869 | 9 2 0 0 | H9N2 |
| Q9Y4L1 | HYOU1 | 931 | 6 2 0 0 | H6N2 |
| Q9Y4L1 | HYOU1 | 931 | 8 2 0 0 | H8N2 |
| Q9Y639 | NPTN | 229 | 7 2 0 0 | H7N2 |
| Q9Y639 | NPTN | 229 | 8 2 0 0 | H8N2 |
| Q9Y639 | NPTN | 229 | 9 2 0 0 | H9N2 |

**Table S3. The quantified 47 de novo N-glycopeptides under NGI-1 treatment**

| **Protein accession number** | **Protein** | **N-glycosylation Sites** | **Glycan (H N S F)** | **Glycan Compositions** | **Mean ratio (NGI trm/DMSO)** | **P-value** | **Alterations in NGI-1 treatment ^△^** |
| --- | --- | --- | --- | --- | --- | --- | --- |
| O14672 | ADA10 | 278 | 8 2 0 0 | H8N2 | 0.72 | 0.9256 | - |
| O14672 | ADA10 | 278 | 9 2 0 0 | H9N2 | 1.39 | 0.4400 | - |
| O14672 | ADA10 | 439 | 8 2 0 0 | H8N2 | 0.39 | 0.5915 | - |
| O75477/O94905 | ERLN1/ERLN2 | 108/106 | 8 2 0 0 | H8N2 | 1.90 | 0.3654 | - |
| O75477/O94905 | ERLN1/ERLN2 | 108/106 | 9 2 0 0 | H9N2 | 1.63 | 0.1165 | - |
| P00533 | EGFR | 352 | 7 2 0 0 | H7N2 | 0.48 | 0.1456 | - |
| P00739/P00738 | HPTR/HPT | 126/184 | 5 4 2 0 | H5N4S2 | 1.70 | 0.6413 | - |
| P05556 | ITB1 | 669 | 6 2 0 0 | H6N2 | 1.63 | 0.5773 | - |
| P05556 | ITB1 | 669 | 8 2 0 0 | H8N2 | 0.35 | 0.4137 | - |
| P05556 | ITB1 | 669 | 9 2 0 0 | H9N2 | 0.66 | 0.5768 | - |
| P11047 | LAMC1 | 1395 | 8 2 0 0 | H8N2 | 2.88 | 0.4406 | - |
| P11279 | LAMP1 | 103 | 7 2 0 0 | H7N2 | 0.45 | 0.1116 | - |
| P13473 | LAMP2 | 356 | 6 2 0 0 | H6N2 | 0.23 | 0.0434 | ↓ |
| P13473 | LAMP2 | 356 | 6 3 1 0 | H6N3S1 | 0.46 | 0.0692 | - |
| P13473 | LAMP2 | 356 | 7 2 0 0 | H7N2 | 0.28 | 0.1595 | - |
| P14625 | ENPL | 217 | 6 2 0 0 | H6N2 | 10.31 | 0.2741 | - |
| P14625 | ENPL | 217 | 7 2 0 0 | H7N2 | 4.98 | 0.3292 | - |
| P14625 | ENPL | 217 | 8 2 0 0 | H8N2 | 3.27 | 0.3960 | - |
| P20645 | MPRD | 57 | 8 2 0 0 | H8N2 | 0.44 | 0.1869 | - |
| P43308 | SSRB | 88 | 7 2 0 0 | H7N2 | 2.47 | 0.2832 | - |
| P43308 | SSRB | 88 | 8 2 0 0 | H8N2 | 3.46 | 0.2308 | - |
| P43308 | SSRB | 88 | 9 2 0 0 | H9N2 | 10.84 | 0.4118 | - |
| P46977 | STT3A | 548 | 7 2 0 0 | H7N2 | 1.49 | 0.3737 | - |
| P46977 | STT3A | 548 | 8 2 0 0 | H8N2 | 5.14 | 0.3165 | - |
| P46977 | STT3A | 548 | 9 2 0 0 | H9N2 | 2.62 | 0.4554 | - |
| P53801 | PTTG | 54 | 7 2 0 0 | H7N2 | 2.13 | 0.5109 | - |
| P55283/P19022 | CADH4/CADH2 | 661/651 | 8 2 0 0 | H8N2 | 0.49 | 0.1440 | - |
| P69849/Q15155/Q5JPE7 | NOMO3/NOMO1/NOMO2 | 369/369/369 | 8 2 0 0 | H8N2 | 0.60 | 0.2473 | - |
| Q4KMQ2 | ANO6 | 361 | 7 2 0 0 | H7N2 | 1.15 | 0.9092 | - |
| Q4KMQ2 | ANO6 | 361 | 8 2 0 0 | H8N2 | 0.71 | 0.6331 | - |
| Q5JRA6 | TGO1 | 246 | 8 2 0 0 | H8N2 | 0.16 | 0.6147 | - |
| Q8N766 | EMC1 | 913 | 9 2 0 0 | H9N2 | 2.29 | 0.3481 | - |
| Q8NBJ4 | GOLM1 | 144 | 8 2 0 0 | H8N2 | 2.27 | 0.4821 | - |
| Q8NFQ8 | TOIP2 | 286 | 8 2 0 0 | H8N2 | 1.30 | 0.3022 | - |
| Q92542 | NICA | 435 | 8 2 0 0 | H8N2 | 3.30 | 0.0465 | ↑ |
| Q96KA5 | CLP1L | 91 | 6 2 0 0 | H6N2 | 0.33 | 0.7656 | - |
| Q9BU23 | LMF2 | 616 | 8 2 0 0 | H8N2 | 3.84 | 0.2590 | - |
| Q9H330 | TM245 | 551 | 8 2 0 0 | H8N2 | 0.31 | 0.5411 | - |
| Q9HD45 | TM9S3 | 174 | 5 4 2 0 | H5N4S2 | 1.59 | 0.4939 | - |
| Q9HDC9 | APMAP | 160 | 6 2 0 0 | H6N2 | 0.84 | 0.5464 | - |
| Q9UHN6 | CEIP2 | 914 | 9 2 0 0 | H9N2 | 0.34 | 0.0049 | ↓ |
| Q9Y4L1 | HYOU1 | 515 | 8 2 0 0 | H8N2 | 1.21 | 0.6364 | - |
| Q9Y4L1 | HYOU1 | 515 | 9 2 0 0 | H9N2 | 1.77 | 0.3830 | - |
| Q9Y4L1 | HYOU1 | 869 | 8 2 0 0 | H8N2 | 2.11 | 0.4325 | - |
| Q9Y4L1 | HYOU1 | 869 | 9 2 0 0 | H9N2 | 2.01 | 0.4781 | - |
| Q9Y639 | NPTN | 229 | 8 2 0 0 | H8N2 | 1.18 | 0.9103 | - |
| Q9Y639 | NPTN | 229 | 9 2 0 0 | H9N2 | 0.70 | 0.0892 | - |

**Table S4. Site-specific glycan profiling of LAMP2 under NGI-1 treatment**

| **Cell line** | **NGI-1 treatment** | **log**_2_Ratio (NGI trm/DMSO) | | | | | | | | | |
| --- | --- | --- | --- | --- | --- | --- | --- | --- | --- | --- | --- |
|  |  | **Asn257** | **Asn275** | | **Asn356** | | | | | | |
|  |  | **H5N4S1F1** | **H5N4S1** | **H6N5S1** | **H5N4F1** | **H5N4S1** | **H6N2** | **H6N3S1** | **H6N5S1** | **H7N2** | **H8N2** |
| **Huh7** | **12h** | -0.576 | -2.352 | -2.408 | -0.548 | -1.319 | -1.343 | -1.394 | -0.741 | -1.568 | -2.138 |
|  | **24h** | -1.060 | -1.351 | -2.918 | -0.858 | -1.534 | -2.752 | -1.917 | -1.918 | -1.092 | -2.384 |
| **HCCLM3** | **12h** | -1.132 | -0.697 | -2.112 | -0.063 | -1.802 | -0.572 | -0.257 | -1.772 | -0.547 | -0.134 |
|  | **24h** | -2.024 | -1.917 | -3.477 | -0.089 | -2.020 | -1.958 | -0.874 | -2.119 | -0.321 | -1.060 |
